# Inhibition of TPL2 by interferon-α suppresses bladder cancer through activation of PDE4D

**DOI:** 10.1186/s13046-018-0971-4

**Published:** 2018-11-27

**Authors:** Zhe Qiang, Zong-yuan Zhou, Ting Peng, Pu-zi Jiang, Nan Shi, Emmanuel Mfotie Njoya, Bahtigul Azimova, Wan-li Liu, Wei-hua Chen, Guo-lin Zhang, Fei Wang

**Affiliations:** 1 Key Laboratory of Natural Medicine and Clinical Translation, Chengdu Institute of Biology, Chinese Academy of Science, Chengdu, China; 2 Key Laboratory of Molecular Biophysics of the Ministry of Education, Hubei Key Laboratory of Bioinformatics and Molecular-imaging, Department of Bioinformatics and Systems Biology, College of Life Science and Technology, Huazhong University of Science and Technology, Wuhan, China; 3 College of Life Sciences, Henan Normal University, Xinxiang, China; 4 Ministry of Education Key Laboratory of Protein Science, School of Life Sciences, Tsinghua University, Beijing, China; 5 Department of Biochemistry, Faculty of Science, University of Yaoundé I, Yaoundé, Cameroon; 6 University of Chinese Academy of Sciences, Beijing, China

**Keywords:** Interferon, TPL2, PDE4D, cAMP, COX-2

## Abstract

**Background:**

Drugs that inhibit the MEK/ERK pathway have therapeutic benefit in bladder cancer treatment but responses vary with patients, for reasons that are still not very clear. Interferon-α (IFN-α) is also used as a therapeutic agent for bladder cancer treatment but the response rate is low. It was found that IFN-α could enhance the cytotoxic effect of MEK inhibition. However, the potential mechanisms of that are still unclear. Understanding of the cross-talk between the IFN-α and MEK/ERK pathway will help enhance the efficacy of IFN-α or MEK inhibitors on bladder cancer.

**Methods:**

Immunoprecipitation and pull-down assay were used to reveal the formation of signaling complex. The protein expressions were detected by western blot and immunohistochemistry. The cAMP level, Phosphodiesterase 4D (PDE4D) activity and Prostaglandin E_2_ (PGE_2_) concentration in cells, serum and tissues were detected by enzyme-linked immunosorbent assay. The role of PDE4D in bladder tumorigenesis in vivo was examined by the xenograft model. Tissue microarray chips were used to investigate the prognostic roles of PDE4D and tumor progression locus 2 (TPL2) in bladder cancer patients.

**Results:**

IFN-α down-regulated the cyclooxygenase-2 (COX-2) expression in bladder cancer cells through the inhibition of TPL2/NF-κB pathway; IFN-α also inhibited COX-2 expression by suppressing cAMP signaling through TPL2-ERK mediated PDE4D activity. Reduction of the intracellular cAMP level by PDE4D potentiated the antitumor effect of IFN-α against bladder cancer in vitro and in vivo. Further analysis of clinical samples indicated that low PDE4D expression and high level of TPL2 phosphorylation were correlated to the development and poor prognosis in bladder cancer patients.

**Conclusions:**

Our data reveal that IFN-α can exert its antitumor effect through a non-canonical JAK-STAT pathway in the bladder cancer cells with low activity of IFN pathway, and the TPL2 inhibition is another function of IFN-α in the context of bladder cancer therapy. The antitumor effects of IFN-α and MEK inhibition also depend on the PDE4D-mediated cAMP level in bladder cancer cells. Suppression of the TPL2 phosphorylation and intracellular cAMP level may be possible therapeutic strategies for enhancing the effectiveness of IFN-α and MEK inhibitors in bladder cancer treatment.

**Electronic supplementary material:**

The online version of this article (10.1186/s13046-018-0971-4) contains supplementary material, which is available to authorized users.

## Background

Bladder cancer is the ninth most common cancer worldwide, particularly in highly developed countries [1]. Compared with non-muscle invasive bladder cancer (NMIBC), the muscle invasive bladder cancer (MIBC) represents a more aggressive cancer type with a mere five-year survival period in < 50% of the cases [2]. Multiple novel druggable target molecules in bladder cancer were identified, and among these, 45% belong to the receptor tyrosine kinase (RTK)-MAPK pathway [3–6]. As a member of the MAPK cascade, TPL2 (also known as COT or MAP3K8) was reported to be a novel therapeutic target in certain inflammatory and cancerous disorders [7]. The TPL2 phosphorylation primarily activates ERK through a MEK-dependent mechanism [8] and is involved in the NF-κB pathway regulation through IκB kinase (IKK) complex [9]. Notably, both these pathways were identified to be associated with the grade, stage, and survival outcome of bladder cancer patients [4, 10]. Therefore, the inhibition of TPL2 activation might improve MIBC treatment; however, further studies are required to understand the underlying mechanisms.

Cyclooxygenase-2 (COX-2) is a key enzyme in prostaglandin E_2_ (PGE_2_) production and COX-2 overexpression is associated with bladder neoplasia development [11]. PGE_2_ level is frequently elevated at the tumor sites [12] and chemotherapy-induced apoptotic cells release PGE_2_, which in turn promotes tumorigenesis and resistance against therapeutic agents in bladder cancer treatment [13]. Inhibition of COX-2-PGE_2_ pathway was reported to reduce drug resistance in xenograft models of urothelial cell carcinoma [10, 14]. In the NMIBC treatment, IFN-α is used clinically in combination with Bacillus Calmette-Guerin (BCG) and currently it is a preventive agent against distant metastases and local recurrence although the response rate of patients is merely 15% [15]. However, the mechanism involved in the poor response of patients towards IFN treatment remains unclear. IFN-α was also found to enhance the efficacy of chemotherapeutic drugs by suppressing the NF-κB activity in advanced renal cell carcinoma [16]. Therefore, IFN-α might suppress COX-2-PGE_2_ pathway through the inhibition of NF-κB activation and this mechanism needs to be further investigated in bladder cancer.

The cAMP is a key second messenger through which PGE_2_ exerts its physiological functions [12]. Studies also showed that cAMP could stimulate the proliferation and cyst formation of renal epithelial cells [17, 18]. In a recent study, IFN-α suppressed cAMP level through MEK/ERK-mediated PDE4 activation and deactivated the suppressive function of human regulatory T cells [19], which was reported previously to reduce the risk of renal cancer progression [20]. Moreover, the down-regulation of PDE4D7 was reported recently to promote the prostate cancer progression through compartmentalization of cAMP [21, 22]. IFN-α/β treatment was also found to strongly enhance the cytotoxic effect of MEK inhibition solely in melanoma cell lines with low activity of IFN pathway [23]. Thus, we aimed to investigate whether the cross-talk between TPL2/MEK/ERK pathway and PDE4D/cAMP signaling mediates the antitumor effect of IFN-α and the potential of these molecules as biomarkers for targeted molecular therapy of bladder cancer.

## Methods

### Cell lines and reagents

T24 and HEK293A cells were obtained from Huaxi Hospital (Chengdu, China) and were authenticated using Short Tandem Repeat (STR) analysis. 5637 cells were purchased from Procell Life Science & Technology Co., Ltd. (Wuhan, China). T24 and 5637 cells were cultured in Roswell Park Memorial Institute Medium (RPMI) 1640 (HyClone) containing 10% (*V*/V) fetal bovine serum (FBS, HyClone). HEK293A cells were cultured in Dulbecco’s modified Eagle’s medium (DMEM) containing 10% (V/V) FBS. All the cell lines were maintained in an incubator with 5% CO_2_ at 37 °C. Forskolin (S2449), PD98059 (S1177), and roflumilast (S2131) were purchased from Selleck Chemicals (Shanghai, China). TPL2 kinase inhibitor (#19710) was purchased from Cayman Chemical (Shanghai, China). Human IFN-α-2a (Z03003) and human EGF (Z02691) were purchased from Genscript., Ltd. (Nanjing, China). Antibodies used in this study as follow: STAT1 (#41462); STAT3 (#41465); p-STAT3 (Tyr705), (#11045); JAK1 (#35530); Tyk2 (#38374); COX-2 (#21679); p- IκBα (Ser32/36), (#11152); IκBα (#29054); CREB (#21052); ERK1/2 (#40901); p-ERK1/2 (Thr202/Tyr204), (#12082); TPL2 (#33235); PDE4D (#23049); IKKα/β (#1057); IFNAR2 (#32426); IFNAR1 (#32400) were purchased from Signalway Antibody, LLC. (Nanjing, China). p-STAT1 (Tyr701), (#9167); p-JAK1 (Tyr1034/1035), (#3331); p-TYK2 (Tyr1054/1055), (#68790); p-TPL2 (Ser400), (#4491); p-CREB (Ser133), (#9198); RACK1 (#5432); P-IKKα/β (Ser176/180), (#2697) were purchased from Cell Signaling Technology, lnc. (Shanghai, China). β-tubulin (#341002) was purchased from Zen Bio Science, Ltd. (Chengdu, China).

### Western blot analysis

Whole cell lysates were extracted using RIPA buffer (Beyotime Biotechnology, China) supplemented with a protease inhibitor cocktail (Sigma, Shanghai, China). The protein concentration was measured using a BCA protein assay kit (Bestbio, Shanghai, China). Cell lysates were performed by sodium dodecyl sulfate-polyacrylamide gel electrophoresis (SDS-PAGE) and protein bands were electrophoretically transferred onto nitrocellulose membranes. After incubation with the primary and secondary antibodies, protein bands were visualized by enhanced-chemiluminescence reaction (Amersham Biosciences, Piscataway, NJ, USA).

### PDE4D overexpression and knockdown

PDE4D overexpression was performed in T24 and 5637 bladder cancer cells using the PDE4D-pReceiver-M11 (or the control) vectors according to the manufacturer’s instructions (Genecopoeia, Rockville, MD, USA). PDE4D knockdown in T24 and 5637 cells was performed using the siRNA sequences targeting PDE4D (stQ0007397) or the non-targeting control siRNA (stQ0007397–1) according to the manufacturer’s instructions (Ribobio, Guangdong, China).

### Cell viability assay

T24 cells were seeded (5 × 10^3^ cells/well) in 96-well plates using 100 μL medium and incubated overnight. The specific drugs used in the particular experiments were diluted in culture medium and added to cells and the plates were incubated for an additional 72 h. The cells of the control group were treated using 0.1% DMSO. Cell proliferation was measured as the absorbance at 450 nm using a cell counting kit-8 (CCK-8) according to the manufacturer’s instructions (Solarbio, China). Experiments were performed in triplicates.

### Trans-well cell migration assay

The migration of T24 and 5637 bladder cancer cells were measured by trans-well assay according to the manufacturer’s instructions (Thermo Fisher, USA). Briefly, T24 and 5637 cells (1 × 10^5^ cells/ml) were added into the trans-wells (100 μL/well) and allowed to migrate under different treatments for 6 h at 37 °C. Cotton swabs were used to remove cells from the upper surface of the trans-wells, and migratory cells attached to the undersurface were stained with crystal violet (0.5%). The numbers of migrated cells (5 different fields per well) were counted using an inverted microscope.

### Enzyme-linked immunosorbent assay (ELISA)

The PGE_2_ levels in cell culture supernatants and serum of mice were estimated according to the manufacturer’s instructions using human PGE_2_ ELISA kit (Invitrogen, USA) and mouse PGE_2_ ELISA kit (Cusabio Technology, USA), respectively.

### cAMP level and PDE4D activity analysis

The cAMP levels in cells and the xenograft tumor tissues were quantified according to the manufacturer’s instructions using a cAMP-Glo™ assay kit (Promega, USA). The enzyme activities of PDE4D isoforms that were immunoprecipitated from cells and xenograft tumor tissues were quantified according to the manufacturer’s instructions using a PDE-Glo™ phosphodiesterase assay kit (Promega).

### Immunoprecipitation and pull-down assay

The extracts of T24 cells and xenograft tumor tissues were precleared with protein A/G agarose beads (Santa Cruz Biotechnology) and incubated with primary antibodies overnight at 4 °C. Later, these samples further incubated with protein A/G agarose beads for 2 h at 4 °C. The immunoprecipitate were suspended in sample buffer and detected by performing western blotting or activity analysis.

### Mouse xenograft model

Female BALB/c (nu/nu) nude mice (aged 5-weeks) were purchased from Dashuo Laboratory Animal Technology, Ltd. (Chengdu, China) and were kept on a 12-h day/night cycle with free access to food and water. All the experiments and procedures were performed in accordance with the National Institutes of Health guide for the care and use of Laboratory animals (NIH Publications No. 8023, revised 1978). T24 or 5637 cells (5 × 10^6^ cells/mouse) were subcutaneously injected into the flanks of mice in serum-free RPMI 1640 (100 μL). The tumor volume was calculated using the formula: volume = 1/2 (length × width^2^). When the tumor volume was approximately 100 to 150 mm^3^, mice were randomly segregated into six groups (seven mice per group) and treated using specific drugs or inhibitors. The tumor size was measured every 3rd day with a caliper. After 28 days (T24 cells) or 24 days (5637 cells), mice were sacrificed to surgically remove tumors and measure the tumor volume and weight. The serum of each mouse was collected to perform PGE_2_ analysis. To determine cAMP level and for PDE4D activity analyses, the lysates of xenograft tumor tissues were extracted using SDS lysis buffer (Beyotime Biotechnology, China). The expression levels of specific proteins in the xenograft tumor tissues were analyzed using tissue microarrays by Outdo Biotech, Ltd. (Shanghai, China).

### Tissue microarray (TMA) and immunohistochemical (IHC) analysis

TMA chips that consisted of the bladder tumor tissue specimens (*n* = 126) and adjacent normal bladder tissue specimens (*n* = 40) were purchased from Outdo Biotech, Ltd. (Shanghai, China). Hematoxylin-eosin (H&E) staining was performed by following the routine method and IHC of TMA chips was performed using primary antibodies against PDE4D (1:150) and pTPL2 (1:150). The assignment of positive-staining score was based on the percentage of positive-staining (0% positive: 0, 1–25% positive: 1, 26–50% positive: 2, 51–75% positive: 3, and 76–100% positive: 4) and staining intensity score was based on the staining intensity (no intensity: 0, weak intensity: 1+, moderate intensity: 2+, and strong intensity: 3+). The final staining index was calculated using the formula: positive-staining score × staining intensity score. These scores were independently determined by two pathologists who were blinded to the clinical and pathological information. IHC staining of TMA using each antibody was performed in a single experiment by including the negative staining control.

### Statistical analysis

The statistical significance of variations among the experimental groups was evaluated using Student t-test and one-way and two-way analyses of variance (ANOVA) tests in the analyses of cAMP levels, PGE_2_ production, cell viability, and PDE4D activity. Data are represented as mean ± standard deviation (SD). Survival analysis was performed using Kaplan-Meier method and compared with the log-rank test. Wilcoxon signed rank test (unpaired comparisons) was used to determine the significant variations among the expressions of particular proteins in the bladder tumor tissues and adjacent bladder tissues. Spearman’s rank correlation coefficient was used to analyze the correlation among the expressions of specific proteins and various clinicopathological features in patients. Data are represented as mean ± standard error of mean (SEM). *P* < 0.05 was considered as statistically significant value. SPSS 13.0 software (SPSS, Chicago, IL, USA) was used to perform survival and correlation analyses, while Prism version 6.07 (GraphPad Software) to perform other analyses.

## Results

### IFN-α suppresses COX-2 expression by inhibition of the TPL2-mediated NF-κB activation and cAMP/CREB pathway

**Fig. 1:**
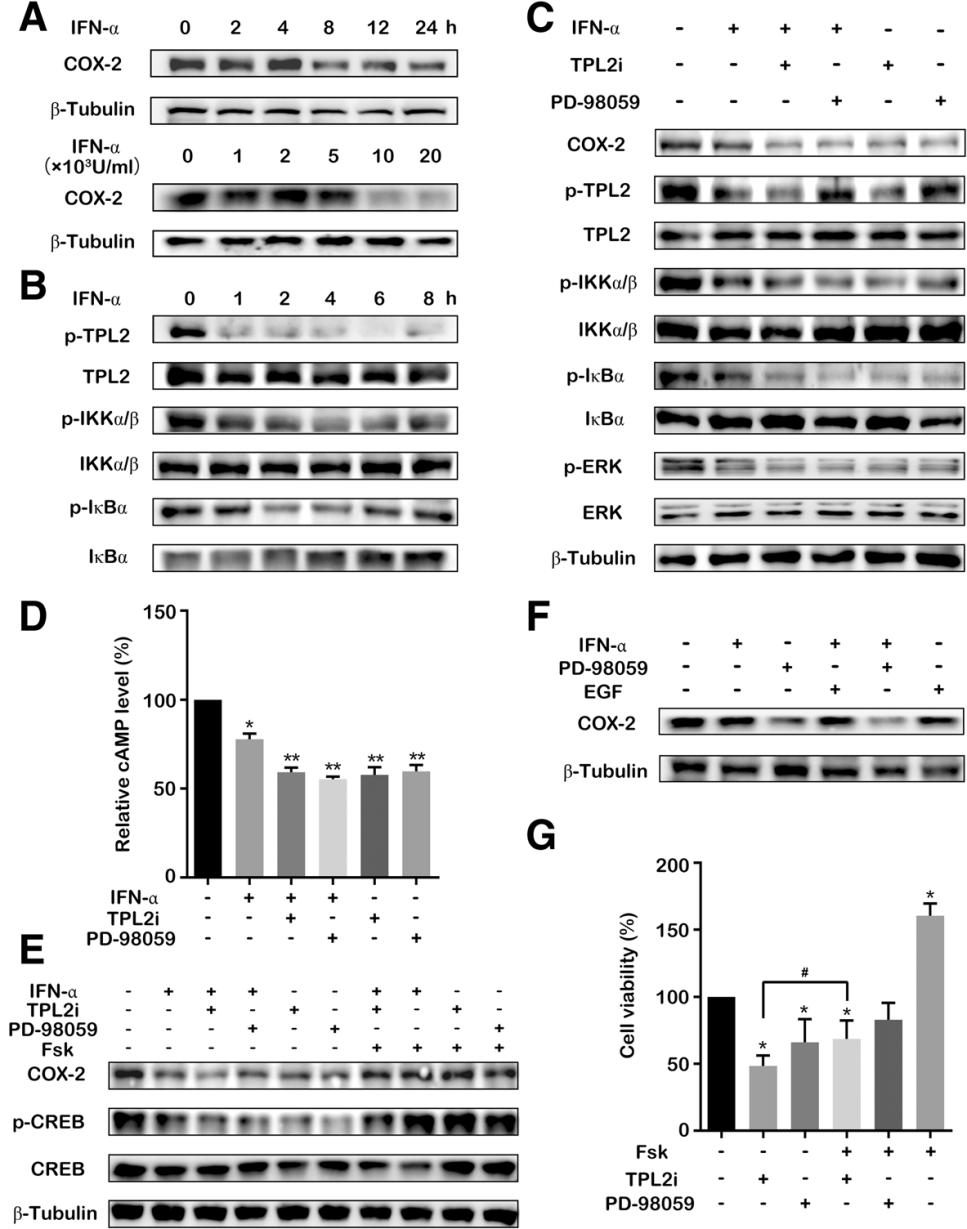
Suppression of COX-2 expression by IFN-α via inhibition of the TPL2 and cAMP/CREB. (**a**) T24 cells were treated with IFN-α (1 × 10^4^ U/mL) for specific time points; or treated using various concentrations of IFN-α for 24 h. The cell lysates were immunoblotted with COX-2 antibody. β-Tubulin staining is shown as a loading control. (**b**) T24 cells were treated with IFN-α (1 × 10^4^ U/mL) for specific time points. The TPL2, p-TPL2, IKKα/β, p- IKKα/β, IκBα and p-IκBα were analyzed by performing western blotting. (**c**) T24 cells were treated with IFN-α (1 × 10^4^ U/ml), TPL2i (2 μM), and PD98059 (40 μM) for 12 h. The COX-2, TPL2, p-TPL2, ERK, p- ERK, IKKα/β, p-IKKα/β, IκBα and p-IκBα were analyzed by performing western blotting. β-Tubulin staining is shown as a loading control. (**d**) The intracellular cAMP level was detected after T24 cells were treated with IFN-α (1 × 10^4^ U/mL), TPL2i (2 μM), and PD98059 (40 μM) for 4 h. (**e**) T24 cells were treated with IFN-α (1 × 10^4^ U/mL), TPL2i (2 μM), PD98059 (40 μM), or forskolin (50 μM) for 24 h. The expression levels of COX-2, CREB, and p-CREB were analyzed by western blotting. The β-tubulin was used as the loading control. (**f**) T24 cells were treated with IFN-α (1 × 10^4^ U/mL), PD98059 (40 μM), and EGF (25 ng/mL) for 12 h. The COX-2 expression was analyzed by performing western blotting. (**g**) Cell viability was detected after T24 cells were treated using forskolin (50 μM), TPL2i (2 μM), and PD98059 (40 μM) for 72 h. Data represent the results of three independent experiments. Error bars indicate mean ± SD. *, *P* < 0.05; **, *P* < 0.01; #, *P* < 0.05 (*t*-test)

COX-2 plays a significant role in the bladder tumorigenesis [11]; however, the effect and mechanism of IFN-α in the regulation of COX-2 expression remains unclear. In this study, IFN-α decreased COX-2 expression in a time- and dose-dependent manner in T24 and 5637 bladder cancer cells (Fig. 1a and Additional file 1: Figure S1). COX-2 is known to be induced by activation of the NF-κB pathway that is regulated by TPL2 in addition to various other factors [9, 24]. Therefore, we examined whether TPL2 mediates the inhibitory effect of IFN-α on NF-κB activation. IFN-α inhibited the phosphorylation of TPL2, ERK, IKK α/β and IκBα and stabilized IκBα expression, indicating that IFN-α decreases COX-2 expression by inhibition of TPL2-NF-κB pathway (Fig. 1b and Additional file 2: Figure S2). To confirm the aforementioned result, bladder cancer cells were treated with TPL2 kinase inhibitor (TPL2i) and MEK inhibitor (PD98059). COX-2 expression and the phosphorylation of IKK α/β and IκBα were inhibited by TPL2i in the presence or absence of IFN-α (Fig. 1c and Additional file 3: Figure S3). Similar results were observed after PD98059 treatment (Fig. 1c). Consistent with a previous report [25], we found that the canonical JAK-STAT signaling in T24 cells was barely affected by IFN-α (Additional file 4: Figure S4A-B), indicating that IFN-α decreased the COX-2 expression through a non-canonical JAK/STAT pathway.

The cAMP/CREB pathway is another main modulator of COX-2 expression [26, 27]. The cAMP level is also regulated by IFN-α-induced MEK/ERK-mediated PDE4 activity [19]. Therefore, we further investigated whether IFN-α decreases the COX-2 expression through TPL2-mediated cAMP/CREB pathway. In T24 cells, IFN-α down-regulated the intracellular cAMP level which was further decreased by the treatment with TPL2i or PD98059 (Fig. 1d). Consistent with the aforementioned result, the CREB phosphorylation was inhibited by IFN-α in the presence or absence of TPL2i or PD98059 accompanied by the down-regulation of COX-2 expression. Conversely, forskolin (a cAMP elevator) counteracted the down-regulation of COX-2 expression induced by IFN-α and TLP2i or PD98059 (Fig. 1e). Furthermore, the reduction of COX-2 expression by IFN-α was abrogated after the treatment with epidermal growth factor (EGF) that is known to activate ERK phosphorylation [28] (Fig. 1f). To determine whether the decrease of intracellular cAMP level inhibits the bladder cancer cell growth, we used TPL2i or PD98059, and forskolin to treat the respective bladder cancer cell groups. TPL2i or PD98059 treatment reduced the bladder cancer cell viability and this reduction was attenuated by forskolin. Furthermore, the cell growth was promoted after the individual treatment of forskolin (Fig. 1g). These data suggested that IFN-α inhibited COX-2 expression through TPL2-mediated inhibition of the NF-κB activation and cAMP/CREB pathway.

### TPL2 regulates the cAMP-hydrolyzing activity of PDE4D at IFNAR2

**Fig. 2:**
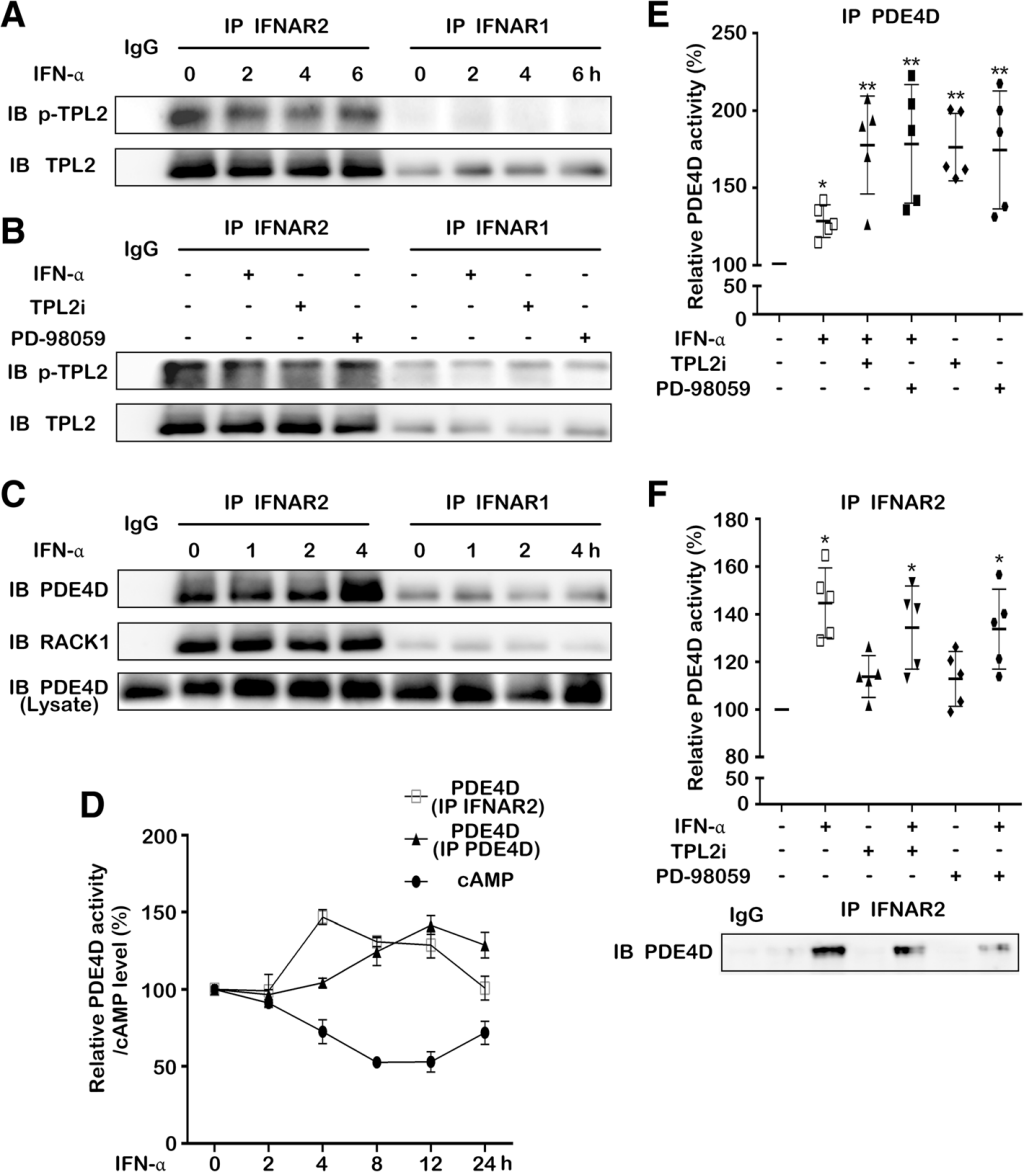
Regulation of PDE4D activity at IFNAR2 by TPL2. (**a**) T24 cells were treated with IFN-α (1 × 10^4^ U/mL) for specific time points. The levels of phosphorylated and total TPL2 bound to IFNAR2 or IFNAR1 were detected by performing western blotting after co-immunoprecipitation using IFNAR2 or IFNAR1 antibodies. (**b**) T24 cells were treated with IFN-α (1 × 10^4^ U/mL), TPL2i (2 μM), and PD98059 (40 μM) for 4 h. The levels of phosphorylated and total TPL2 bound to IFNAR2 or IFNAR1 were detected by performing western blotting after co-immunoprecipitation using IFNAR2 or IFNAR1 antibodies. (**c**) T24 cells were treated with IFN-α (1 × 10^4^ U/mL) for specific time points. The levels of RACK1 and PDE4D that interacted with IFNAR2 or IFNAR1 were detected after co-immunoprecipitation using IFNAR2 or IFNAR1 antibodies. (**d**) T24 cells were treated with IFN-α (1 × 10^4^ U/mL) for specific time points. The intracellular cAMP level, activity of total PDE4D, and activity of PDE4D that interacted with IFNAR2 were detected after co-immunoprecipitation using IFNAR2 or PDE4D antibodies. (**e**) T24 cells were treated with IFN-α (1 × 10^4^ U/mL), TPL2i (2 μM), and PD98059 (40 μM) for 4 h. The activity of total PDE4D was detected after immunoprecipitation using PDE4D antibody. (**f**) T24 cells were treated with IFN-α (1 × 10^4^ U/mL), TPL2i (2 μM), and PD98059 (40 μM) for 4 h. The PDE4D that interacted with IFNAR2 and their activity were detected after co-immunoprecipitation using IFNAR2 antibody. Data represent the results of five independent experiments. Error bars indicate mean ± SD. *, *P* < 0.05; **, *P* < 0.01 (*t*-test)

In order to understand the mechanism involved in the regulation of TPL2 by IFN-α, we examined the interaction between TPL2 and IFNAR by performing co-immunoprecipitation. TPL2 was found to interact with IFNAR2 (but not IFNAR1) and this interaction was barely affected by IFN-α (Fig. 2a). IFN-α and TPL2i suppressed the level of pTPL2 that interacted with IFNAR2, whereas they did not affect the interaction between IFNAR2 and un-phosphorylated TPL2 (Fig. 2b). We previously found that RACK1 orchestrates the localization of PDE4D and protein kinase A (PKA) at IFNAR2 [29]. Therefore, we further determined the function of PDE4D in the IFN-α induced cAMP suppression. The interaction between the PDE4D and IFNAR2 was examined by performing co-immunoprecipitation. Unlike TPL2, PDE4D was recruited to IFNAR2 through RACK1 after the IFN-α treatment (Fig. 2c and Additional file 5: Figure S5A-C). Additionally, the activity of PDE4D that interacted with IFNAR2 was increased after IFN-α treatment, followed by the increase in the activity of total intracellular PDE4D. Consequently, the intracellular cAMP level was observed to be steadily reduced (Fig. 2d). These results indicated that IFN-α suppresses the cAMP level by enhancing the PDE4D activity through a dynamic interaction between PDE4D and IFNAR2. To further examine the role of TPL2-MEK/ERK pathway in the regulation of PDE4D activity, cells were treated with IFN-α and/or TPL2i or PD98059. The treatments using the individual inhibitors and combination of IFN-α and inhibitors exhibited a stronger effect on the enhancement of total intracellular PDE4D activity compared to individual IFN-α treatment (Fig. 2e). However, the variation in the PDE4D activity that leads to the interaction with IFNAR2 was very low after the treatment using individual TPL2i or PD98059 (Fig. 2f). This suggested that IFNAR2 does not recruit the reactivated PDE4D that is induced by TPL2-MEK inhibition. Collectively, these data indicated that IFN-α suppresses the cAMP level through TPL2-MEK/ERK-mediated PDE4D activity at IFNAR2.

### Induction of PDE4D expression by roflumilast synergizes with IFN-α activity to reduce intracellular cAMP level

**Fig. 3:**
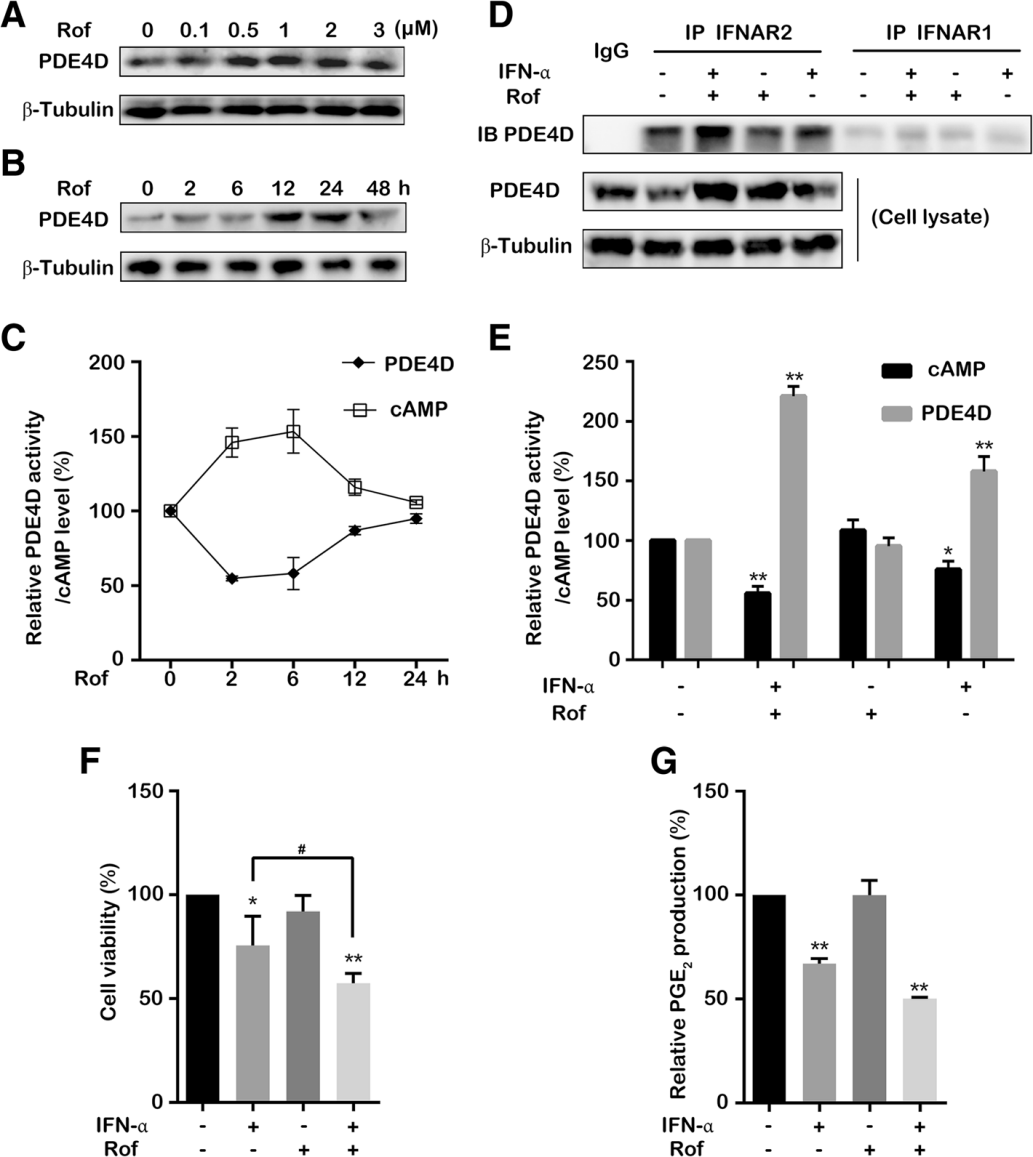
Induction of PDE4D by roflumilast potentiates anti-proliferation effect of IFN-α in vitro. (**a**) T24 cells were treated using the specific concentrations of roflumilast for 24 h. (**b**) T24 cells were treated with roflumilast (1 μM) for specific time points. (**c**) T24 cells were treated with roflumilast (1 μM) for specific time points. Intracellular cAMP levels and activity of immunoprecipitated PDE4D were detected. (**d**) T24 cells were treated with IFN-α (1 × 10^4^ U/mL) and roflumilast (1 μM) either individually or in combination for 24 h. The level of PDE4D that interacted with IFNAR2 or IFNAR1 was detected by performing western blotting after co-immunoprecipitation using IFNAR2 or IFNAR1 antibodies. The expression levels of total PDE4D and β-tubulin in the cell lysates were used as loading control. (**e**) T24 cells were treated with IFN-α (1 × 10^4^ U/mL) and roflumilast (1 μM) either individually or in combination for 24 h. The intracellular cAMP level and activity of total PDE4D were detected after immunoprecipitation using PDE4D antibody. (**f**, **g**) The cell viability (**f**) and PGE_2_ production (**g**) were detected after T24 cells were treated with IFN-α (1 × 10^4^ U/mL) and roflumilast (1 μM) either individually or in combination for 72 h. Data represent the results of three independent experiments. Error bars indicate mean ± SD. *, *P* < 0.05; **, *P* < 0.01; #, *P* < 0.05 (*t*-test)

The expression of PDE4 isoforms (PDE4A-D) is induced by PDE4 inhibitors [30]. Recently, roflumilast was reported to induce the PDE4B/4D expression in human respiratory tract epithelial cells [31]. We attempted to determine whether roflumilast induces the PDE4D expression in bladder cancer cells. The PDE4D expression was up-regulated by roflumilast treatment in a dose-dependent manner (Fig. 3a). Additionally, the PDE4D expression was significantly increased during 12–24 h after roflumilast treatment (Fig. 3b). However, the concomitant intracellular PDE4D activity and cAMP level reverted to their normal levels after 12 h and subsequently remained unchanged (Fig. 3c). These results indicated that the increased PDE4D by roflumilast did not reduce the intracellular cAMP level continuously. These observations prompted us to speculate whether IFN-α enhances the PDE4D activity that is induced by roflumilast and further reduces the intracellular cAMP level. The result showed that more PDE4D were recruited at IFNAR2 after the combination treatment of IFN-α and roflumilast (Fig. 3d). Moreover, the treatment using the combination of IFN-α with roflumilast caused a stronger effect on the enhancement of total PDE4D activity and reduction of intracellular cAMP level compared to individual IFN-α or roflumilast treatment (Fig. 3e). cAMP was reported to stimulate the proliferation in renal epithelial cells [17, 18]. Therefore, we further investigated whether the synergistic reduction of cAMP level by the combinations of IFN-α and inhibitors of TPL2-MEK-PDE4D pathway have effects on the proliferation of bladder cancer cells. The treatment using the combinations of IFN-α and inhibitors (TPL2i, PD98059 or roflumilast) exhibited a stronger inhibitory effect on the viability of bladder cancer cells than the individual IFN-α treatment (Fig. 3f and Additional file 6: Figure S6A-B). PDE4D overexpression and knockdown were then performed to further examine the role of PDE4D in the regulation of proliferation and migration in bladder cancer cells. The results showed that the overexpression of PDE4D inhibited the proliferation of bladder cancer cells and the knockdown of PDE4D, conversely, promoted the cell growth (Additional file 6: Figure S6C-E). The knockdown of PDE4D also increased the migration of bladder cancer cells and IFN-α (or TPL2 inhibitor) reduced the number of migrated cells only when the PDE4D protein was overexpressed (Additional file 6: Figure S6F-G). We then observed the morphological alterations of cells after the knockdown of PDE4D and found that only the 5637 bladder cancer cells became irregular in shape and extended tentacles (Additional file 6: Figure S6H). Furthermore, roflumilast was found to enhance the inhibitory effect of IFN-α on PGE_2_ production, which plays an important role in tumorigenesis of bladder cancer (Fig. 3g). Taken together, these data suggested that the induction of PDE4D expression by roflumilast synergized with IFN-α activity to reduce the intracellular cAMP level and potentiates the antiproliferative effect of IFN-α on bladder cancer cells.

### Roflumilast potentiates the anti-tumor effect of IFN-α in vivo

**Fig. 4:**
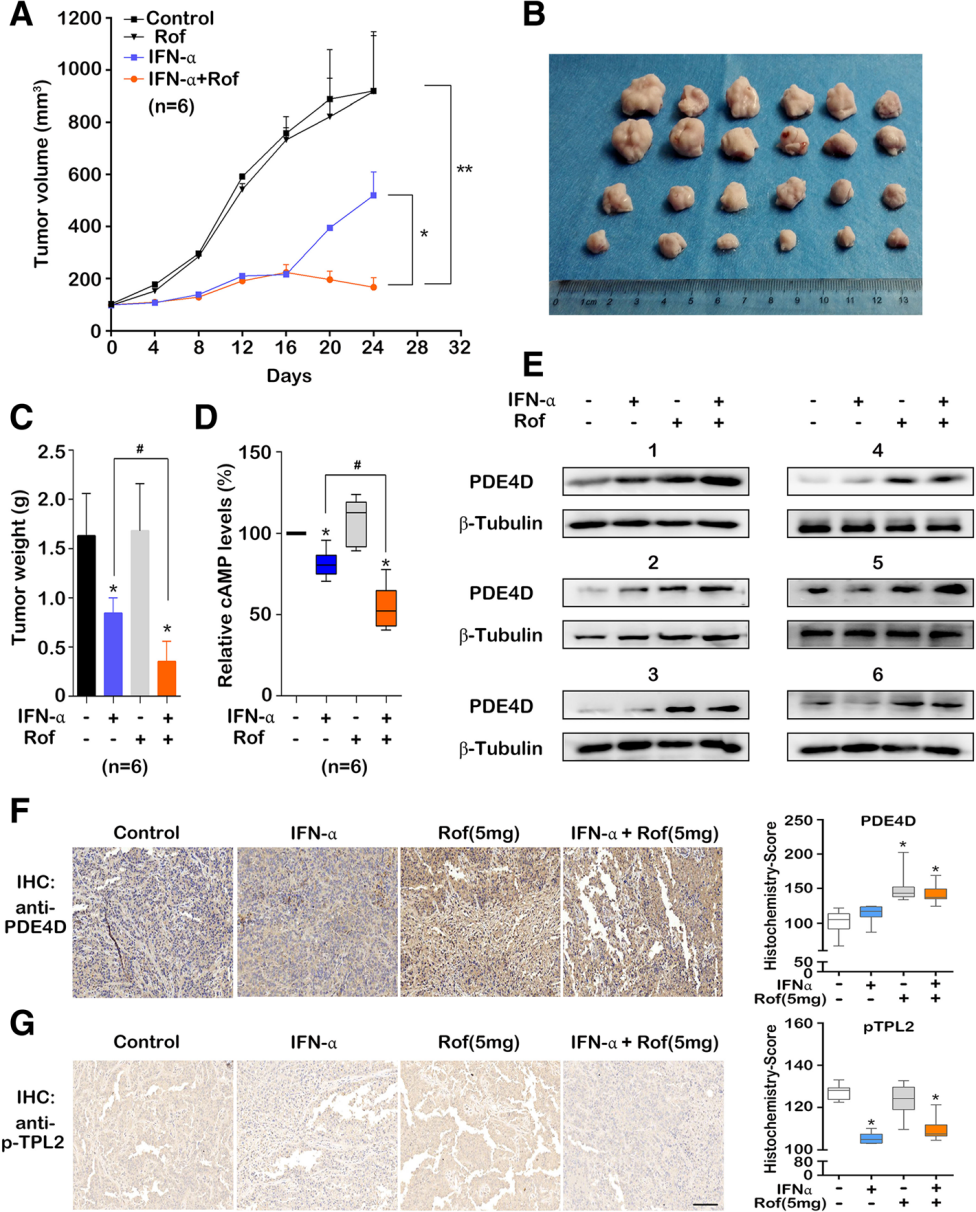
Roflumilast potentiated the anti-tumor effect of IFN-α in vivo. 5637 cells (5 × 10^6^ cells/mouse) were subcutaneously injected into BALB/c nude mice. When the tumor size was ~ 100 mm^3^, mice were treated with phosphate buffered saline (control), roflumilast (5 mg/kg/day, oral administration), and IFN-α (1 × 10^4^ U/mouse/2 days, intraperitoneal injection) either individually or in combination for 24 days before sacrifice. The tumor volumes were measured every 4 days. (**a**) The tumor growth curves of all the treatment groups. Each data point indicates the mean of tumor volume (*n* = 6 per group). (**b**) Image of the tumors in all the treatment groups. (**c**) The tumor weights in all the treatment groups (n = 6 per group). (**d**, **e**) The lysis of tumor tissues in all treatment groups were used to detected the cAMP levels (**d**) and expressions of PDE4D (**e**). (**f**, **g**) IHC and difference analyses of PDE4D (**f**) and pTPL2 (**g**) expressions (Histochemistry-Score) among the T24 tumor tissues of the indicated groups. Error bars indicate mean ± SD (*n* = 7). *, *P* < 0.05; **, *P* < 0.01; #, *P* < 0.05 (*t*-test and Mann-Whitney test)

To investigate whether roflumilast potentiates the anti-tumor effect of IFN-α in vivo, we used a tumor xenograft model by injecting human 5637 bladder cancer cells into BALB/c nude mice (see materials and methods). The combination treatment of IFN-α and roflumilast (5 mg/kg/day) drastically suppressed the tumor growth compared to individual IFN-α treatment (Fig. 4a-c). We further investigated whether roflumilast (5 mg/kg/day) potentiates the anti-tumor effect of IFN-α through the cAMP reduction. The lysates obtained from xenograft tumor tissues were used to detect the cAMP level. The cAMP level in tumor lysates was significantly reduced after the combination treatment of IFN-α and roflumilast compared to individual IFN-α or roflumilast treatment (Fig. 4d). Next, the PDE4D expression in tumor tissues was evaluated by western blotting. IFN-α did not affect the PDE4D expression; however, roflumilast treatment induced the PDE4D expression when used individually or in combination with IFN-α (Fig. 4e). The results from T24 tumor xenograft model also showed that the combination treatment of IFN-α and roflumilast (5 mg/kg/day) potentiated the anti-tumor effect of IFN-α through the cAMP reduction (Additional file 7: Figure S7A-D). Consistent with the in vitro result, the PDE4D activity was increased in the individual IFN-α treatment, and in the treatment of roflumilast combined with IFN-α further enhanced the PDE4D activity (Additional file 7: Figure S7E). Moreover, we examined the PGE_2_ production in the mice serum. IFN-α as well as roflumilast individual treatment exhibited inhibitory effects on the PGE_2_ production; IFN-α and roflumilast combination treatment further reduced the PGE_2_ production than either of the individual treatments (Additional file 7: Figure S7F). The PDE4D expression and TPL2 phosphorylation in T24 tumor tissues were evaluated by immunohistochemistry (IHC). The PDE4D expression increased when roflumilast was used individually or in combination with IFN-α (Fig. 4f). Furthermore, high pTPL2 level was observed in tumor tissues and was inhibited by IFN-α. However, roflumilast did not affect both the TPL2 phosphorylation and IFN-α induced inhibition of TPL2 phosphorylation (Fig. 4g). These data suggested that the induction of PDE4D expression by roflumilast potentiated the anti-tumor effect of IFN-α through the elevated PDE4D expression and reduction of intracellular cAMP.

### PDE4D expression and TPL2 phosphorylation levels are correlated with the human bladder cancer development

**Fig. 5:**
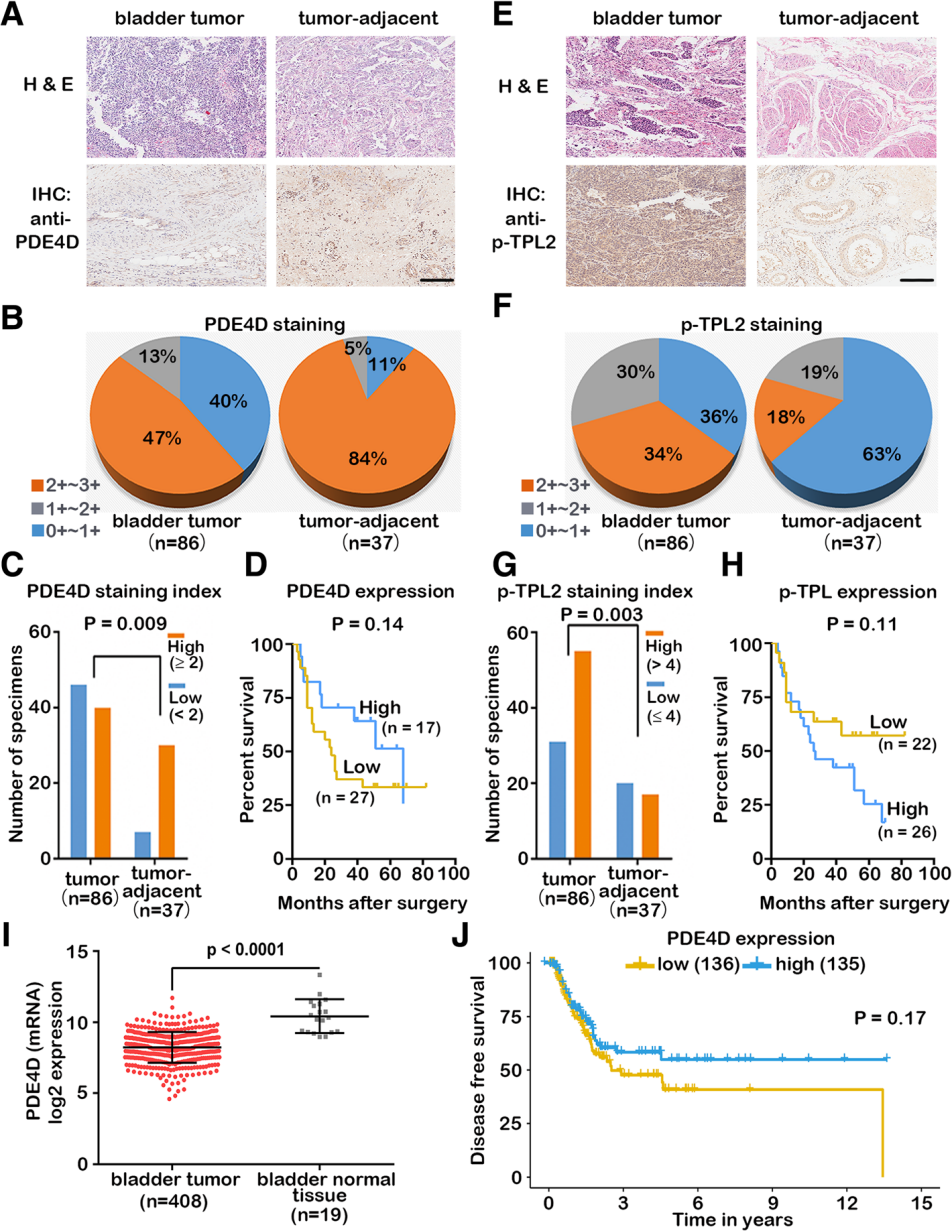
Correlations of PDE4D expression and TPL2 phosphorylation with human MIBC development. (**a**) H&E and IHC staining of PDE4D in the representative bladder tumor tissues and the adjacent normal bladder tissues. (Scale bar: 200 μm). (**b**) Statistical data of PDE4D staining in the bladder tumor tissues and adjacent normal bladder tissues. (**c**) Because all specimens had the same positive-staining scores of PDE4D, we used the staining intensity score to replace the staining index. All the specimens were segregated into two groups based on their staining index (lower expression < staining index 2; higher expression ≥ staining index 2) and compared to observe the variations. (**d**) Kaplan-Meier survival curves based on the PDE4D expression levels to demonstrate the prognostic importance of PDE4D. (**e**) H&E and IHC staining of p-TPL2 in the representative bladder tumor tissues and adjacent normal bladder tissues. (Scale bar: 200 μm). (**f**) Statistical data of p-TPL2 staining in the bladder tumor tissues and adjacent normal bladder tissues. (**g**) All specimens were segregated into two groups based on their staining index (high: ≥ 4 and low: < 4) and compared to observe the variations. (**h**) Kaplan-Meier survival curves based on the p-TPL2 levels to demonstrate the prognostic importance of pTPL2. (**i**, **j**) The data derived from TCGA database were analyzed and PDE4D mRNA levels were significantly down-regulated in the bladder tumor compared to the bladder normal tissue (**i**) and correlated with the poor prognosis (**j**). Error bars indicate mean ± SEM. Statistical significances of differences between experimental groups were evaluated using the Wilcoxon signed rank test (**c** and **g**), unpaired Wilcoxon test (**i**), and log-rank test (**d**, **h**, and **j**). *P* < 0.01 was considered as statistically significant value

To explore the clinical importance of the TPL2 phosphorylation and PDE4D expression levels in human bladder cancer development, the tissue microarray chips that consisted of MIBC specimens (*n* = 126) were used to perform IHC analysis. The bladder cancer sections and the adjacent bladder normal tissues were obtained from the patients who underwent surgical resection. The H&E staining was performed using the routine method (Additional file 8: Figure S8) and the IHC staining results were analyzed using the staining index (see materials and methods). Statistically, the PDE4D expression was found to be significantly lower in the bladder tumor tissues than that in adjacent normal bladder tissues (*P* = 0.009) (Fig. 5a-c, Additional file 9: Figure S9, Additional file 10: Table S1), and the low PDE4D expression was positively correlated to the poor prognosis (Fig. 5d).

Unlike PDE4D expression, the level of TPL2 phosphorylation was found to be significantly higher in the bladder tumor tissues than that in the adjacent bladder normal tissues (*P* = 0.003) (Fig. 5e-g, Additional file 11: Figure S10, Additional file 10: Table S1), and the high level of pTPL2 was positively correlated to poor prognosis (Fig. 5h). The survival curves that correspond to each of the clinicopathologic features were also analyzed as the basic information about the tissue microarray chips (Additional file 12: Figure S11). Additionally, the low PDE4D expression and high pTPL2 levels were found to positively correlate with the age and TNM stages of bladder cancer patients (Additional file 10: Table S2). To further validate our TMA-IHC results, we analyzed the data of bladder cancer patients derived from The Cancer Genome Atlas (TCGA) database. In TCGA data, a significantly low (cancer vs. normal) PDE4D expression was observed in bladder cancer patients (Fig. 5i) and correlated with their poor prognosis (Fig. 5j). Moreover, the data also revealed that the PDE4B expression was significantly lower in the bladder cancer tissues than that in the bladder normal tissues (Additional file 13: Figure S12B); however, variations were not observed in the cases of PDE4A and PDE4C expressions (Additional file 13: Figure S12A and C). The data derived from Oncomine database indicated similar results of PDE4D expression (Additional file 14: Figure S13A-D); however, regarding the un-phosphorylated TPL2 expression, variations were not observed between the bladder mucosal and bladder tumors (Additional file 14: Figure S13E). Together, these results suggest that the low PDE4D expression and high pTPL2 levels are correlated to the MIBC development and poor prognosis in MIBC patients.

## Discussion

The NF-κB/COX-2 pathway is activated through IκB kinase (IKK) complex [9] and the overexpression of COX-2 plays a significant role in the bladder tumorigenesis [11]. IKK activity is required to activate the TPL2-ERK axis [32], however, the serine^400^ phosphorylation of TPL2 also activates the IKK complex through NF-κB-inducing kinase (NIK) [9, 24]. Our results showed that IFN-α did not affect the constitutive TPL2 expression but inhibited the serine^400^ phosphorylation of TPL2 and subsequent IKKα/β phosphorylation, suggesting that IFN-α might inhibit the IKKα/β activation through TPL2. Notably, the serine^400^ residue of TPL2 is phosphorylated in a protein kinase B (AKT) dependent manner [24] and the mutational activation of phosphatidylinositol 3-kinase (PI3K)/AKT pathway is common in bladder cancer [33]. This is another probable reason for the TPL2 activation and COX-2 overexpression in bladder cancer.

The intracellular cAMP was reported to promote the proliferation in renal epithelial cells and stimulates the cyst formation in diseased kidney cells [17, 18]. This is supported by our observation that an increase of cAMP level promotes the bladder cancer cell proliferation. The modulation of NF-κB pathway by cAMP/CREB is highly dependent on cell-type and -condition [27]. The cAMP-activated IKK causes NF-κB activation [34, 35] and PKA (a main effector of cAMP) also activates NF-κB by the destabilization of protein phosphatase 2C beta (PP2Cβ; a negative regulator of NF-κB) [36]. CREB is another major transcriptional factor involved in the regulation of COX-2 expression and CREB activation is regulated in a TPL2-dependent manner [37]. We found that IFN-α also suppressed COX-2 expression by reducing the intracellular cAMP level through TPL2/ERK-mediated PDE4D activity in bladder cancer cells. IFN-α is clinically used in bladder cancer but the underlying mechanism of resistance against IFN-α therapy remains unclear [15]. Consistent with a previous report [25], we also found that IFN-α barely affect JAK-STAT pathway in bladder cancer cells, which suggest that IFN-α might exert antitumor effect by inhibiting COX-2 expression independent of canonical JAK/STAT pathway. Thus, our findings are helpful to understand the antitumor effect of type I IFNs in cells with low activity of IFN pathway and could provide novel insight into the oncogenic role of TPL2 in bladder cancer.

The majority of bladder cancers are highly dependent on ERK that is activated by the alterations of FGFR, MAPK/MEK or Notch pathways [3, 5, 6]. Although the inhibitors of FGFR or MEK indicate promising improvement in bladder cancer treatment, responses vary with patients and the reasons are still not very clear [3, 4]. Furthermore, ERK activation was found to phosphorylate PDE4D at the catalytic region and thus causes the inhibition of cAMP-hydrolyzing activity [38]. Here we showed that the PDE4D activity was repressed by the constitutive activation of TPL2/ERK in bladder cancer cells and the antitumor effect of IFN-α-induced TPL2/ERK inhibition partially depended on the PDE4D-mediated cAMP level. This is further supported by the observation that IFN-α/β enhances the cytotoxic efficiency of MEK inhibitors in melanoma cell lines with low IFN activity [23]. Our finding provides a probable explanation for the response heterogeneity of MEK inhibition in cancer treatment [3] because the regulatory effect of MEK inhibition on PDE4D activity largely depends on the cell-type/environment [8, 39] and finally leads to the different change of cAMP level.

The recruitment of PDE4D to specific intracellular sites is important for the cAMP compartmentalization [39] and ERK was found to interact with IFNAR2 [40]. In this study, we demonstrated that a signaling complex formed by TPL2, RACK1, and PDE4D at IFNAR2 facilitated IFN-α to inhibit TPL2 phosphorylation and enhance PDE4D activity, which in turn suppressed the NF-κB activation and intracellular cAMP level. RACK1 is a signaling scaffold protein and it binds with IFNAR2 to mediate the recruitment and activation of STAT1 protein by IFN [41]. RACK1 also specifically recruits PDE4D through a helical domain but does not affect the PDE4D activity [42]. Our results indicated that RACK1 bound to IFNAR2 and recruited PDE4D after the IFN-α stimulation, which facilitated IFN-α to enhance the PDE4D activity through TPL2/ERK. It suggested that the formation of a signaling complex at IFNAR2 might generate a local compartment of low cAMP concentration and assist IFN-α to exert its function. This provides new insight into the observations that the cAMP counteracts apoptosis and growth inhibition induced by IFN-α [43]. Furthermore, RACK-1 was found to modulate NF-κB activation [44], indicating that RACK-1 might also involve in IFN-α induced IKK inhibition.

The expression of PDE4 isoforms could be induced by cAMP elevator including PDE4 inhibitors [30, 31]. Roflumilast is an FDA-approved PDE4 inhibitor that is orally administered. Recently, roflumilast was reported to induce PDE4B and PDE4D expression in human epithelial cells [31]. In this study, we found that the induction of PDE4D expression by roflumilast synergized with IFN-α to reduce the cAMP level and potentiated the antiproliferation effect of IFN-α on bladder cancer in both the cell lines and mice xenograft model. The roflumilast-induced PDE4D did not alter the intracellular cAMP level after 12 h in bladder cancer cells, reinforcing the notion that the PDE4D activity is stringently regulated by compartmentalization in cells [38, 39, 45]. Moreover, both the IFN-α and roflumilast were found to inhibit the PGE_2_ production in mice serum. This observation is consistent with the findings that IFN-α or roflumilast inhibit the NF-κB activity and other inflammatory factors [16, 46].

The downregulation of PDE4D expression was found recently to increase the proliferation of prostate cancer cells and associated with the progression of prostate cancer [21, 22]. In this study, lower expression of PDE4D and higher TPL2 phosphorylation were found in the bladder tumor tissues than that in the adjacent normal tissues and correlated with poor prognosis. However, the total TPL2 expression did not vary between the bladder tumor and normal tissues. This suggested that the low PDE4D expression and high level of TPL2 phosphorylation might synergistically induce the cAMP level and promote MIBC development. Because the high level of TPL2 phosphorylation probably induces the COX-2 expression and activates the MEK/ERK pathway to increase cAMP level through the inhibition of PDE4D activity. Considering the important role of PDE4D in the downstream of TPL2-MEK/ERK pathway [19, 39], PDE4D expression might be a prognostic marker in bladder cancer patients with an aberrant MAPK activation.

## Conclusions

**Fig. 6:**
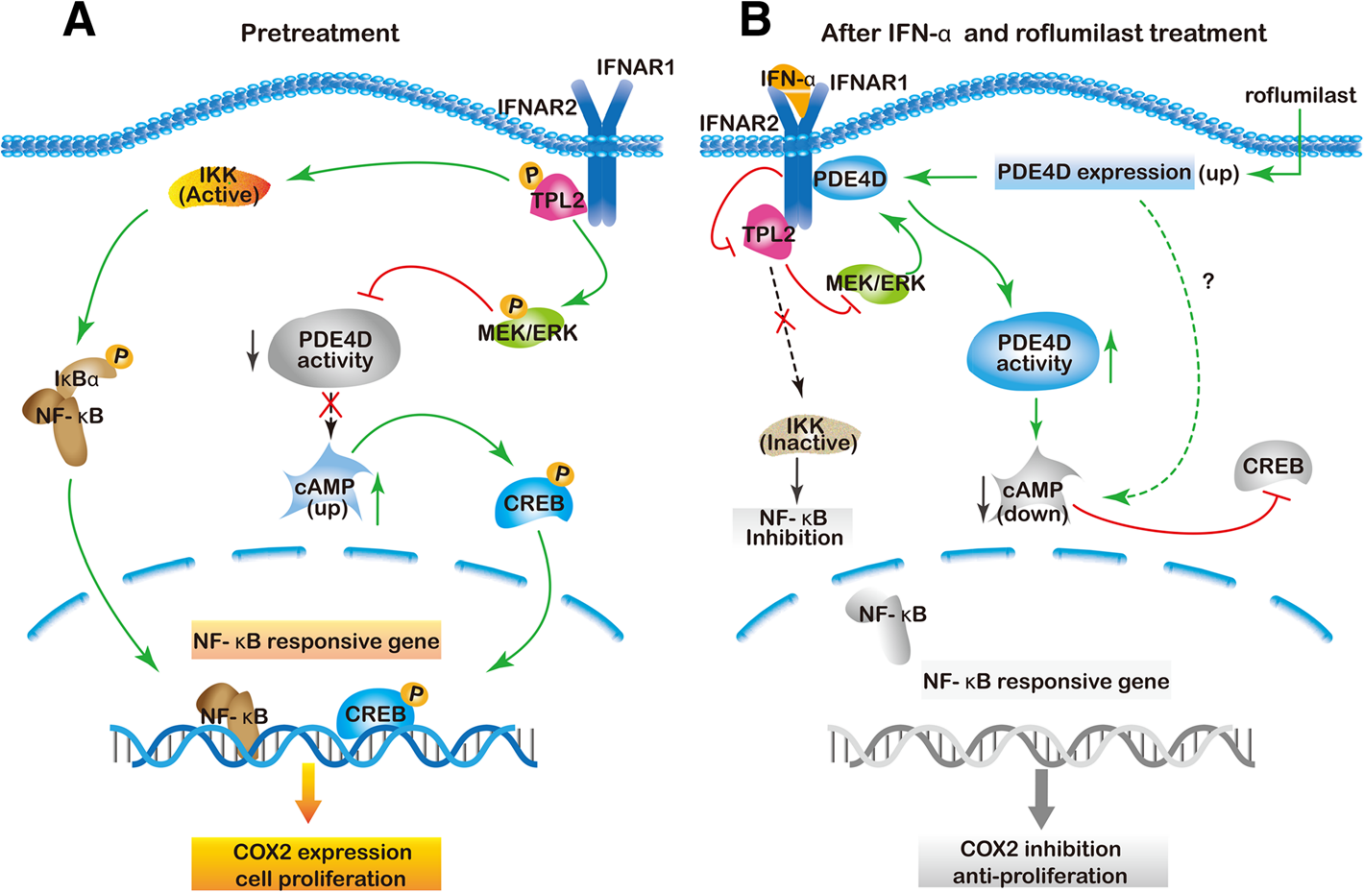
Synergistic antitumor effect of IFN-α and roflumilast on MIBC. (**a**) In MIBC, TPL2 is phosphorylated and activates IKK complexes. Therefore, NF-κB is activated and results in COX-2 overexpression that promotes the MIBC development. Moreover, TPL2 induces COX-2 expression by the enhancement of cAMP/CREB signaling through ERK-mediated inhibition of PDE4D activity. (**b**) IFN-α-induced TPL2 inhibition leads to down-regulation of COX-2 expression and exerts the anti-tumor effect in MIBC treatment. PDE4D induction by roflumilast synergizes with IFN-α activity to inhibit COX-2 expression through the reduction of cAMP level and potentiates the anti-tumor effect of IFN-α on MIBC

In summary, we found that IFN-α exerted anti-tumor effect on bladder cancer cells through the inhibition of TPL2-NF/κB-COX2 pathway and TPL2-ERK-PDE4D mediated cAMP signaling (Fig. 6). The molecular basis is a signaling complex that formed by TPL2, RACK1, and PDE4D at IFNAR2. Reduction of the intracellular cAMP level by PDE4D potentiated the antitumor effect of IFN-α against bladder cancer in vitro and in vivo. These data provide a probable explanation for the response heterogeneity of MEK/ERK inhibition in cancer treatment because the regulatory effect of MEK/ERK inhibition on PDE4D activity largely depends on the cell-type/environment and finally leads to the different changes of cAMP level. Further analysis of clinical samples indicated that low PDE4D expression and high level of TPL2 phosphorylation were also correlated to the development and poor prognosis in bladder cancer patients. This study reveals the novel regulatory effects of TPL2 and PDE4D on the antitumor efficacy of IFN-α and MEK inhibitors in bladder cancer treatment. Pharmaceutical inhibition of TPL2 phosphorylation or reduction of intracellular cAMP level may help develop new therapeutic strategies to enhance the efficacy of IFN-α and MEK inhibitors in bladder cancer treatment.

## Additional files

Additional file 1: **Figure S1.** IFN-α down-regulates COX-2 expression in a time- and dose-dependent manner. **(A)** 5637 cells were treated with IFN-α (2 × 10^4^ U/mL) and the COX-2 expression level was estimated at specific time points. **(B)** 5637 cells were treated with various concentrations of IFN-α for 24 h and the COX-2 expression level was estimated. (PDF 118 kb)

Additional file 2: **Figure S2.** IFN-α inhibits the phosphorylation of ERK in bladder cancer cells. **(A)** 5637 cells were treated with IFN-α (2 × 10^4^ U/mL) for specific time points. **(B)** 5637 cells were treated with various concentrations of IFN-α for 24 h. In both cases, p-ERK and ERK levels were analyzed by performing western blotting. (PDF 107 kb)

Additional file 3: **Figure S3.** IFN-α down-regulates COX-2 expression by inhibition of TPL2. 5637 cells were treated with IFN-α (2 × 10^4^ U/mL), TPL2i (2 μM), and PD98059 (40 μM) either individually or in combination for 24 h. COX-2, p-TPL2, and TPL2 expression levels were analyzed by performing western blotting. β-Tubulin was used as loading control. (PDF 109 kb)

Additional file 4: **Figure S4.** IFN-α inhibits COX-2 expression through non-canonical JAK-STAT signaling. **(A)** T24 cells were treated with IFN-α (1 × 10^4^ U/mL) for 2 h and the expression levels of pJAK1, JAK1, pTyk2, Tyk2, pSTAT1, STAT1, pSTAT3, and STAT3 were estimated at specific time points. **(B)** T24 cells were treated by IFNα (1 × 10^4^ U/ml) and/or JAK kinase inhibitor for 24 h. The expression of COX-2 was analyzed by western blotting. The β-Tubulin was detected as loading control. (PDF 152 kb)

Additional file 5: **Figure S5.** The interaction of RACK1 and PDE4D in vitro. **(A)** The combination of His-PDE4D and GST-RACK1was incubated for 6 h at 4 °C with end-over-end mixing. Pull-down and western blotting were performed to detect the interaction between PDE4D and RACK1. **(B)** HEK293A cell lysates were incubated with GST-RACK1 for 6 h at 4 °C with end-over-end mixing. In HEK293A cell extracts, the PDE4D levels that interacted with GST-RACK1 were pulled down using the glutathione-agarose beads and were detected by performing western blotting. **(C)** HEK293A cell lysates were incubated with His-PDE4D for 6 h at 4 °C with end-over-end mixing. In HEK293A cell extracts, the RACK1 levels that interacted with His-PDE4D were pulled down using the His-tag purification beads and were detected by performing western blotting. GST proteins, His-tag purification beads, or glutathione-agarose beads were individually used with cell lysates as the control group. (PDF 307 kb)

Additional file 6: **Figure S6.** The effects of TPL2-PDE4D pathway on the proliferation, migration and morphology in T24 and 5637 cells. **(A, B)** The cell viability was detected after 5637 cells were treated with IFN-α (1 × 10^4^ U/mL) and/or TPL2i (2 μM), PD98059 (40 μM), roflumilast (1 μM) for 72 h. **(C)** The effects of IFN-α (1 × 10^4^ U/mL) and TPL2i (2 μM) on proliferation after the overexpression or knockdown of PDE4D in T24 and 5637 cells. **(D, E)** The overexpression **(D)** and knockdown **(E)** of PDE4D protein were analyzed by western blotting in T24 and 5637 cells. **(F, G)** The migration of T24 and 5637 cells was analyzed by trans-well assay after the indicated treatments. **(H)** The morphological changes in 5637 cells after the knockdown of PDE4D protein. Cells became irregular in shape and extended tentacles. Data represent the results of three independent experiments. Error bars indicate mean ± SD. *, *P* < 0.05; **, *P* < 0.01; #, *P* < 0.05 (*t*-test). (PDF 357 kb)

Additional file 7: **Figure S7.** Roflumilast potentiated the anti-tumor effect of IFN-α in vivo. T24 cells (5 × 10^6^ cells/mouse) were subcutaneously injected into BALB/c nude mice. When the tumor size was ~ 150 mm^3^, mice were treated with phosphate buffered saline (control), roflumilast (75 μg/kg/day or 5 mg/kg/day, oral administration), and IFN-α (1 × 10^4^ U/mouse/2 days, intraperitoneal injection) either individually or in combination for 28 days before sacrifice. The tumor volumes were measured every 4 days. **(A)** Images of the representative tumors. **(B)** The tumor growth curves of all the treatment groups. Each data point indicates the mean of tumor volume (*n* = 7 per group). **(C)** The tumor weights in all the treatment groups (n = 7 per group). **(D)** cAMP levels in tumor tissues of indicated treatment groups. **(E)** The activity of immunoprecipitated PDE4D obtained from tumor tissues of indicated treatment groups. **(F)** PGE_2_ concentrations in mice serums of indicated treatment groups. Error bars indicate mean ± SD (*n* = 6). *, *P* < 0.05; **, *P* < 0.01; #, *P* < 0.05 (*t*-test and Mann-Whitney test). (PDF 226 kb)

Additional file 8: **Figure S8.** **(A-B)** Hematoxylin and eosin (H&E) staining images of two tissue microarray chips (No. HBlaU060CS01 **[A]** and No. HBlaU066Su01**[B]**). (PDF 345 kb)

Additional file 9: **Figure S9.** **(A-B)** Immunohistochemistry images of two tissue microarray chips (No. HBlaU060CS01 **[A]** and No. HBlaU066Su01**[B]**) for PDE4D expression in the bladder tumor tissues and adjacent normal bladder tissues. (PDF 306 kb)

Additional file 10: **Table S1.** The differences analysis of the PDE4D and the p-TPL2 expressions between in bladder cancer tissues and in adjacent normal tissues. **Table S2.** Statistical analysis of the correlations among PDE4D expression, p-TPL2 expression and clinicopathological parameters. (PDF 110 kb)

Additional file 11: **Figure S10.** **(A-B)** Immunohistochemistry images of two tissue microarray chips (No. HBlaU060CS01 **[A]** and No. HBlaU066Su01**[B]**) for p-TPL2 expression in the bladder tumor tissues and adjacent normal bladder tissues. (PDF 321 kb)

Additional file 12: **Figure S11.** The survival curves of patients that correspond to each of the clinicopathologic features. All data were obtained from the tissue microarray chips that were used in this study. The survival curves were calculated by the Kaplan-Meier method and analyzed by the log-rank test. (PDF 316 kb)

Additional file 13: **Figure S12.** The relationships of bladder cancer with the expression of PDE4 family members. **(A-C)** Compared with PDE4A and PDE4C, the mRNA levels of PDE4B showed a significant down-regulation in bladder tumor than in bladder normal tissue. The data were analyzed by unpaired Wilcoxon test for significance. Values of *P* < 0.01 were considered statistically significant. All RNA-seq data were obtained from The Cancer Genome Atlas. (https://cancergenome.nih.gov/). (PDF 317 kb)

Additional file 14: **Figure S13.** The relationships of bladder cancer with the expression of PDE4 family members and TPL2. **(A-D)** The mRNA levels of PDE4D showed the most significant down-regulation in muscle-invasive bladder cancer (MIBC) when compared with the other three PDE4 family members (PDE4A, 4B and 4C). **(E)** No significant differences of total TPL2 mRNA levels were found among bladder mucosa, NMIBC and MIBC. All data were obtained from the Oncomine database (Lee Bladder Dataset; J Clin Oncol 2010/06/01) and analyzed by unpaired Wilcoxon test. Values of *P* < 0.01 were considered statistically significant. (PDF 226 kb)

## Abbreviations

BCG: Bacillus Calmette-Guerin
cAMP: Cyclic adenosine monophosphate
COX-2: Cyclooxygenase-2
CREB: cAMP-response element binding protein
ERK: Extracellular signal-regulated kinases
FGFR3: Fibroblast growth factor receptor 3
IFN-α: Interferon-α,
IKK: IκB kinase
MAPK: Mitogen-activated protein kinase
MEK, also known as MAPKK: Mitogen-activated protein kinase kinase
MIBC: Muscle invasive bladder cancer
NF-κB: Nuclear factor kappa-light-chain-enhancer of activated B cells
NIK: NF-κB-inducing kinase
NMIBC: Non-muscle invasive bladder cancer
PDE4D: Phosphodiesterase 4D
PGE_2_: Prostaglandin E_2_
AKT: Protein kinase B
PI3K: Phosphatidylinositol 3-kinase
RACK1: Receptor for activated C kinase 1
RTK: Receptor tyrosine kinase
TPL2: Tumor progression locus 2
IFNAR2: Type I interferon receptor 2
IFNAR1: Type I interferon receptor 1
Rof: Roflumilast
TPL2i: TPL2 inhibitor
