# Supplementary material for: Inhibition of TPL2 by interferon-α suppresses bladder cancer through activation of PDE4D
Source: J Exp Clin Cancer Res. 2018 Nov 27;37:288. doi: 10.1186/s13046-018-0971-4 (PMC6260752; doi:10.1186/s13046-018-0971-4)
Supplement: Supplementary file 1 — Figure S1. IFN-α down-regulates COX-2 expression in a time- and dose-dependent manner. (A) 5637 cells were treated with IFN-α (2 × 10^4^ U/mL) and the COX-2 expression level was estimated at specific time points. (B) 5637 cells were treated with various concentrations of IFN-α for 24 h and the COX-2 expression level was estimated. (PDF 118 kb) [file 13046_2018_971_MOESM1_ESM.pdf]

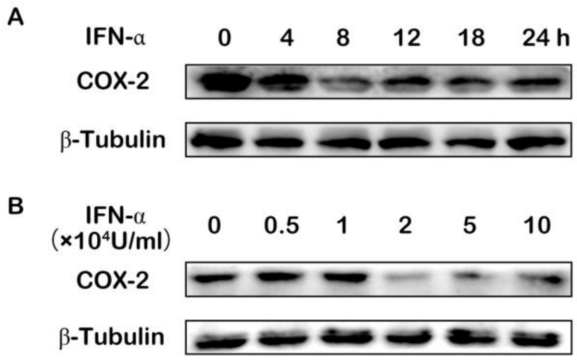

Figure S1. IFN-α down-regulates COX-2 expression in a time- and dose-dependent manner. **(A)** 5637 cells were treated with IFN-α (2 × 10^4 U/mL) and the COX-2 expression level was estimated at specific time points. **(B)** 5637 cells were treated with various concentrations of IFN-α for 24 h and the COX-2 expression level was estimated.
