# Supplementary material for: Inhibition of TPL2 by interferon-α suppresses bladder cancer through activation of PDE4D
Source: J Exp Clin Cancer Res. 2018 Nov 27;37:288. doi: 10.1186/s13046-018-0971-4 (PMC6260752; doi:10.1186/s13046-018-0971-4)
Supplement: Supplementary file 3 — Figure S3. IFN-α down-regulates COX-2 expression by inhibition of TPL2. 5637 cells were treated with IFN-α (2 × 10^4^ U/mL), TPL2i (2 μM), and PD98059 (40 μM) either individually or in combination for 24 h. COX-2, p-TPL2, and TPL2 expression levels were analyzed by performing western blotting. β-Tubulin was used as loading control. (PDF 109 kb) [file 13046_2018_971_MOESM3_ESM.pdf]

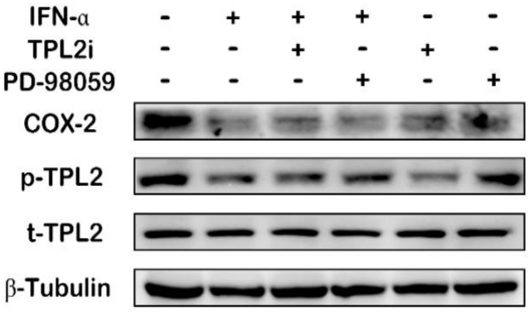

Figure S3: IFN-α down-regulates COX-2 expression by inhibition of TPL2. 5637 cells were treated with IFN-α (2 × 10^4 U/mL), TPL2i (2 μM), and PD98059 (40 μM) either individually or in combination for 24 h. COX-2, p-TPL2, and TPL2 expression levels were analyzed by performing western blotting. β-Tubulin was used as loading control.
