# Supplementary material for: Inhibition of TPL2 by interferon-α suppresses bladder cancer through activation of PDE4D
Source: J Exp Clin Cancer Res. 2018 Nov 27;37:288. doi: 10.1186/s13046-018-0971-4 (PMC6260752; doi:10.1186/s13046-018-0971-4)
Supplement: Supplementary file 4 — Figure S4. IFN-α inhibits COX-2 expression through non-canonical JAK-STAT signaling. (A) T24 cells were treated with IFN-α (1 × 10^4^ U/mL) for 2 h and the expression levels of pJAK1, JAK1, pTyk2, Tyk2, pSTAT1, STAT1, pSTAT3, and STAT3 were estimated at specific time points. (B) T24 cells were treated by IFNα (1 × 10^4^ U/ml) and/or JAK kinase inhibitor for 24 h. The expression of COX-2 was analyzed by western blotting. The β-Tubulin was detected as loading control. (PDF 152 kb) [file 13046_2018_971_MOESM4_ESM.pdf]

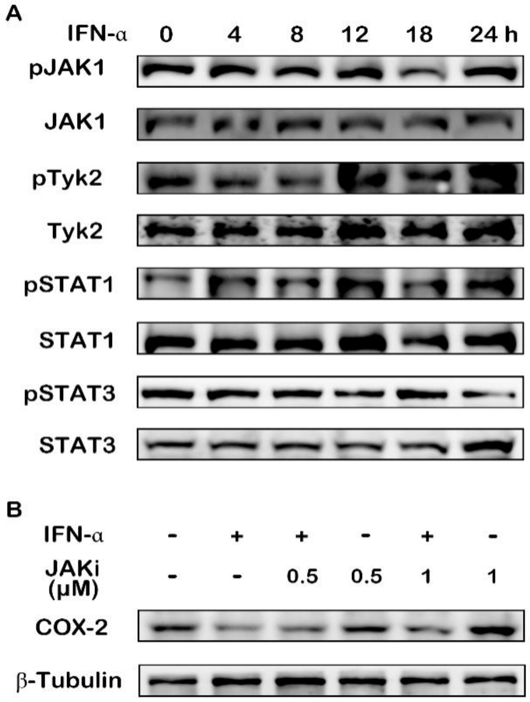

Figure S4: IFN-α inhibits COX-2 expression through non-canonical JAK-STAT signaling. **(A)** T24 cells were treated with IFN-α (1×10^4 U/mL) for 2 hours and the expression levels of pJAK1, JAK1, pTyk2, Tyk2, pSTAT1, STAT1, pSTAT3, and STAT3 were estimated at specific time points. **(B)** T24 cells were treated by IFNα (1×10^4 U/ml) and/or JAK kinase inhibitor for 24 hours. The expression of COX-2 was analyzed by western blotting. The β-Tubulin was detected as loading control.
