# Supplementary material for: Inhibition of TPL2 by interferon-α suppresses bladder cancer through activation of PDE4D
Source: J Exp Clin Cancer Res. 2018 Nov 27;37:288. doi: 10.1186/s13046-018-0971-4 (PMC6260752; doi:10.1186/s13046-018-0971-4)
Supplement: Supplementary file 5 — Figure S5. The interaction of RACK1 and PDE4D in vitro. (A) The combination of His-PDE4D and GST-RACK1was incubated for 6 h at 4 °C with end-over-end mixing. Pull-down and western blotting were performed to detect the interaction between PDE4D and RACK1. (B) HEK293A cell lysates were incubated with GST-RACK1 for 6 h at 4 °C with end-over-end mixing. In HEK293A cell extracts, the PDE4D levels that interacted with GST-RACK1 were pulled down using the glutathione-agarose beads and were detected by performing western blotting. (C) HEK293A cell lysates were incubated with His-PDE4D for 6 h at 4 °C with end-over-end mixing. In HEK293A cell extracts, the RACK1 levels that interacted with His-PDE4D were pulled down using the His-tag purification beads and were detected by performing western blotting. GST proteins, His-tag purification beads, or glutathione-agarose beads were individually used with cell lysates as the control group. (PDF 307 kb) [file 13046_2018_971_MOESM5_ESM.pdf]

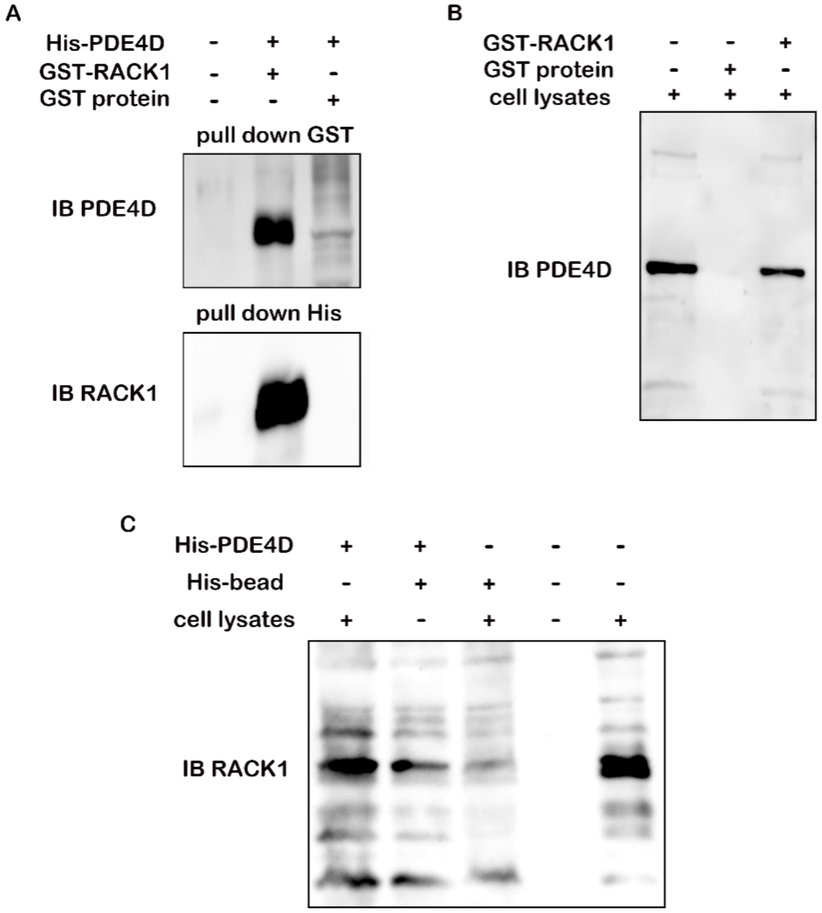

Figure S5: The interaction of RACK1 and PDE4D *in vitro*. **(A)** The combination of His-PDE4D and GST-RACK1was incubated for 6 h at 4 °C with end-over-end mixing. Pull-down and western blotting were performed to detect the interaction between PDE4D and RACK1. **(B)** HEK293A cell lysates were incubated with GST-RACK1 for 6 h at 4 °C with end-over-end mixing. In HEK293A cell extracts, the PDE4D levels that interacted with GST-RACK1 were pulled down using the glutathione-agarose beads and were detected by performing western blotting. **(C)** HEK293A cell lysates were incubated with His-PDE4D for 6 h at 4 °C with end-over-end mixing. In HEK293A cell extracts, the RACK1 levels that interacted with His-PDE4D were pulled down using the His-tag purification beads and were detected by performing western blotting. GST proteins, His-tag purification beads, or glutathione-agarose beads were individually used with cell lysates as the control group.
