# Supplementary material for: Inhibition of TPL2 by interferon-α suppresses bladder cancer through activation of PDE4D
Source: J Exp Clin Cancer Res. 2018 Nov 27;37:288. doi: 10.1186/s13046-018-0971-4 (PMC6260752; doi:10.1186/s13046-018-0971-4)
Supplement: Supplementary file 6 — Figure S6. The effects of TPL2-PDE4D pathway on the proliferation, migration and morphology in T24 and 5637 cells. (A, B) The cell viability was detected after 5637 cells were treated with IFN-α (1 × 10^4^ U/mL) and/or TPL2i (2 μM), PD98059 (40 μM), roflumilast (1 μM) for 72 h. (C) The effects of IFN-α (1 × 10^4^ U/mL) and TPL2i (2 μM) on proliferation after the overexpression or knockdown of PDE4D in T24 and 5637 cells. (D, E) The overexpression (D) and knockdown (E) of PDE4D protein were analyzed by western blotting in T24 and 5637 cells. (F, G) The migration of T24 and 5637 cells was analyzed by trans-well assay after the indicated treatments. (H) The morphological changes in 5637 cells after the knockdown of PDE4D protein. Cells became irregular in shape and extended tentacles. Data represent the results of three independent experiments. Error bars indicate mean ± SD. *, P < 0.05; **, P < 0.01; #, P < 0.05 (t-test). (PDF 357 kb) [file 13046_2018_971_MOESM6_ESM.pdf]

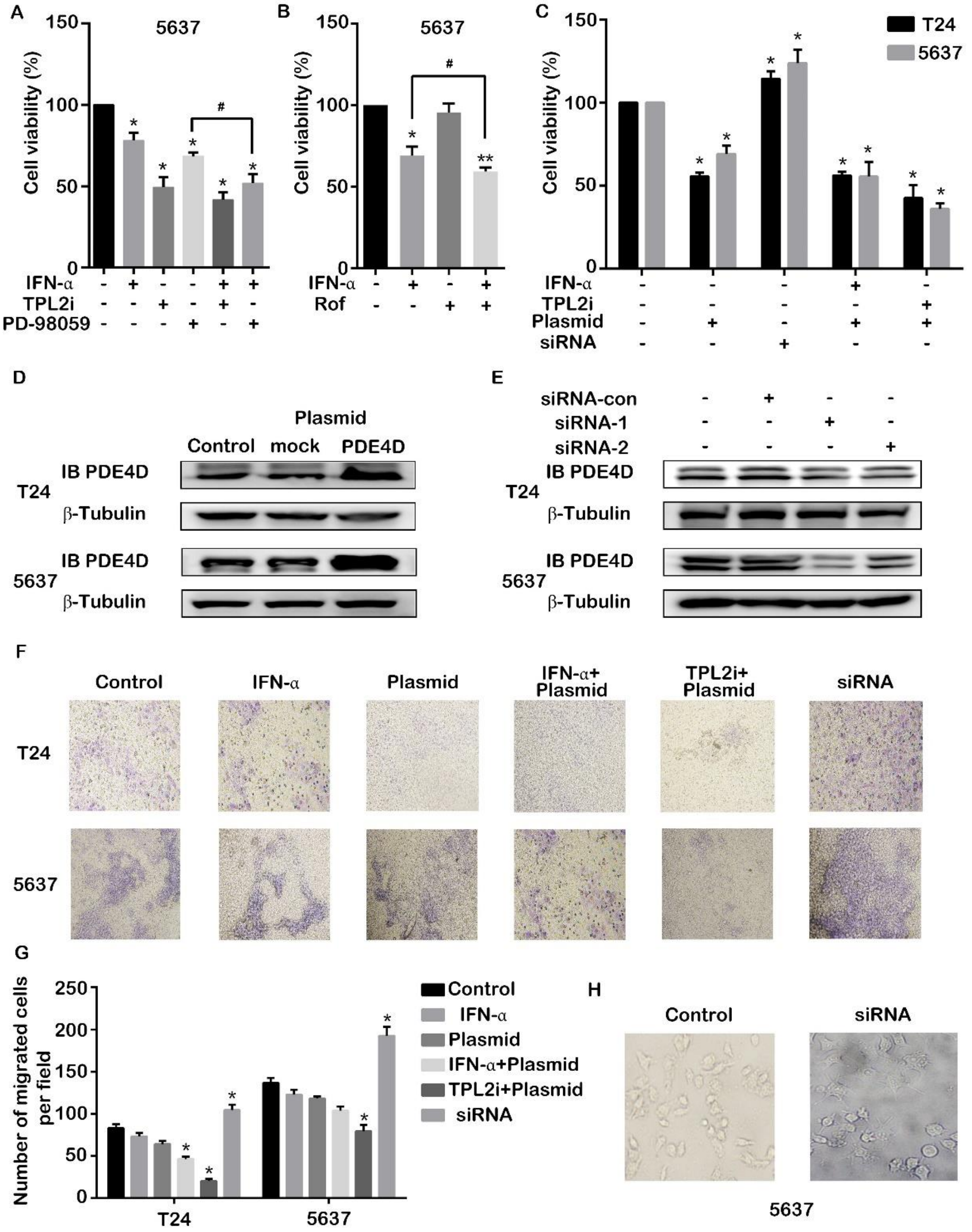

Figure S6: The effects of TPL2-PDE4D pathway on the proliferation, migration and morphology in T24 and 5637 cells. **(A, B)** The cell viability was detected after 5637 cells were treated with IFN-α (1×10^4 U/mL) and/or TPL2i (2 μM), PD98059 (40 μM), roflumilast (1 μM) for 72 h. **(C)** The effects of IFN-α (1×10^4 U/mL) and TPL2i (2 μM) on proliferation after the overexpression or knockdown of PDE4D in T24 and 5637 cells. **(D, E)** The overexpression **(D)** and knockdown **(E)** of PDE4D protein were analyzed by western blotting in T24 and 5637 cells. **(F, G)** The migration of T24 and 5637 cells was analyzed by trans-well assay after the indicated treatments. **(H)** The

morphological changes in 5637 cells after the knockdown of PDE4D protein. Cells became irregular in shape and extended tentacles. Data represent the results of three independent experiments. Error bars indicate mean ± SD. *, $P < 0.05$; **, $P < 0.01$; #, $P < 0.05$ (*t*-test).
