# Supplementary material for: Inhibition of TPL2 by interferon-α suppresses bladder cancer through activation of PDE4D
Source: J Exp Clin Cancer Res. 2018 Nov 27;37:288. doi: 10.1186/s13046-018-0971-4 (PMC6260752; doi:10.1186/s13046-018-0971-4)
Supplement: Supplementary file 7 — Figure S7. Roflumilast potentiated the anti-tumor effect of IFN-α in vivo. T24 cells (5 × 10^6^ cells/mouse) were subcutaneously injected into BALB/c nude mice. When the tumor size was ~ 150 mm3, mice were treated with phosphate buffered saline (control), roflumilast (75 μg/kg/day or 5 mg/kg/day, oral administration), and IFN-α (1 × 10^4^ U/mouse/2 days, intraperitoneal injection) either individually or in combination for 28 days before sacrifice. The tumor volumes were measured every 4 days. (A) Images of the representative tumors. (B) The tumor growth curves of all the treatment groups. Each data point indicates the mean of tumor volume (n = 7 per group). (C) The tumor weights in all the treatment groups (n = 7 per group). (D) cAMP levels in tumor tissues of indicated treatment groups. (E) The activity of immunoprecipitated PDE4D obtained from tumor tissues of indicated treatment groups. (F) PGE2 concentrations in mice serums of indicated treatment groups. Error bars indicate mean ± SD (n = 6). *, P < 0.05; **, P < 0.01; #, P < 0.05 (t-test and Mann-Whitney test). (PDF 226 kb) [file 13046_2018_971_MOESM7_ESM.pdf]

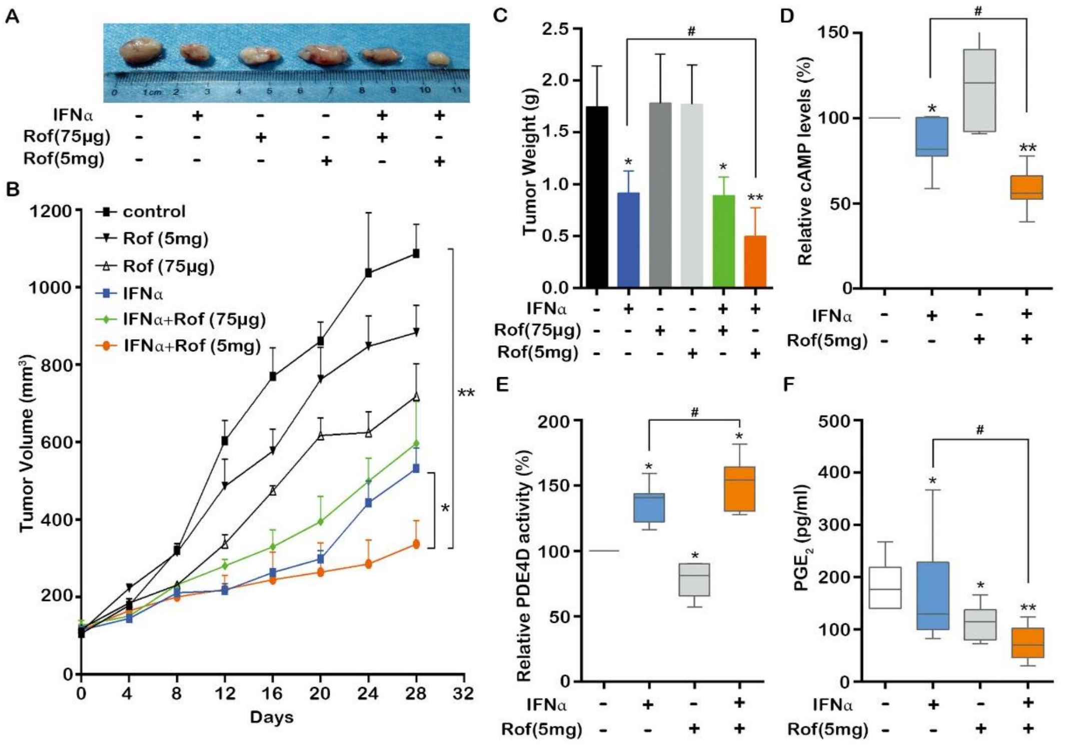

Figure S7: Roflumilast potentiated the anti-tumor effect of IFN-α *in vivo*. T24 cells (5 × 10^6 cells/mouse) were subcutaneously injected into BALB/c nude mice. When the tumor size was ~150 mm^3, mice were treated with phosphate buffered saline (control), roflumilast (75 μg/kg/day or 5 mg/kg/day, oral administration), and IFN-α (1 × 10^4 U/mouse/2 days, intraperitoneal injection) either individually or in combination for 28 days before sacrifice. The tumor volumes were measured every 4 days. **(A)** Images of the representative tumors. **(B)** The tumor growth curves of all the treatment groups. Each data point indicates the mean of tumor volume (n = 7 per group). **(C)** The tumor weights in all the treatment groups (n = 7 per group). **(D)** cAMP levels in tumor tissues of indicated treatment groups. **(E)** The activity of immunoprecipitated PDE4D obtained from tumor tissues of indicated treatment groups. **(F)** PGE_2 concentrations in mice serums of indicated treatment groups. Error bars indicate mean ± SD (n = 6). *, $P < 0.05$; **, $P < 0.01$; #, $P < 0.05$ (*t*-test and Mann-Whitney test).
