# Supplementary material for: Inhibition of TPL2 by interferon-α suppresses bladder cancer through activation of PDE4D
Source: J Exp Clin Cancer Res. 2018 Nov 27;37:288. doi: 10.1186/s13046-018-0971-4 (PMC6260752; doi:10.1186/s13046-018-0971-4)
Supplement: Supplementary file 8 — Figure S8. (A-B) Hematoxylin and eosin (H&E) staining images of two tissue microarray chips (No. HBlaU060CS01 [A] and No. HBlaU066Su01[B]). (PDF 345 kb) [file 13046_2018_971_MOESM8_ESM.pdf]

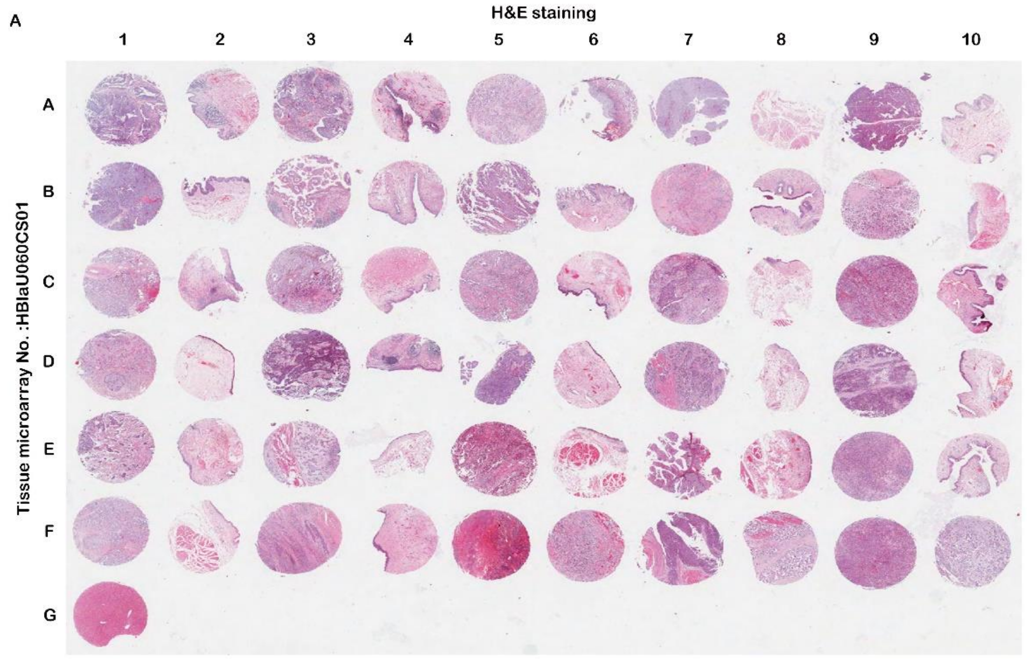

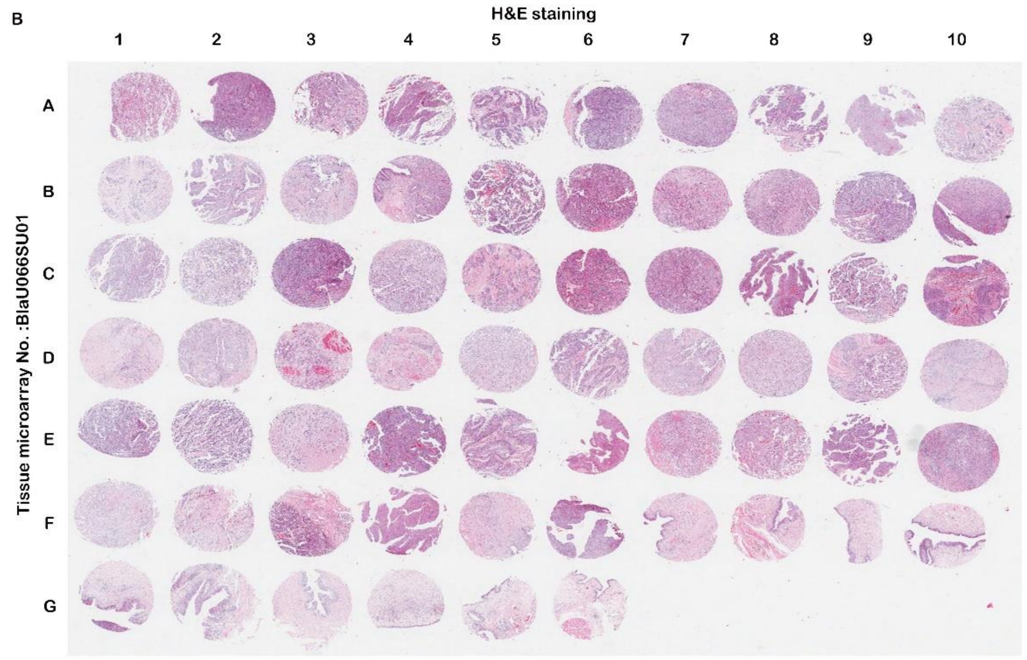

Figure S8: (A-B) Hematoxylin and eosin (H&E) staining images of two tissue microarray chips (No. HBlaU060CS01 **[A]** and No. HBlaU066Su01**[B]**).
