# Supplementary material for: Inhibition of TPL2 by interferon-α suppresses bladder cancer through activation of PDE4D
Source: J Exp Clin Cancer Res. 2018 Nov 27;37:288. doi: 10.1186/s13046-018-0971-4 (PMC6260752; doi:10.1186/s13046-018-0971-4)
Supplement: Supplementary file 9 — Figure S9. (A-B) Immunohistochemistry images of two tissue microarray chips (No. HBlaU060CS01 [A] and No. HBlaU066Su01[B]) for PDE4D expression in the bladder tumor tissues and adjacent normal bladder tissues. (PDF 306 kb) [file 13046_2018_971_MOESM9_ESM.pdf]

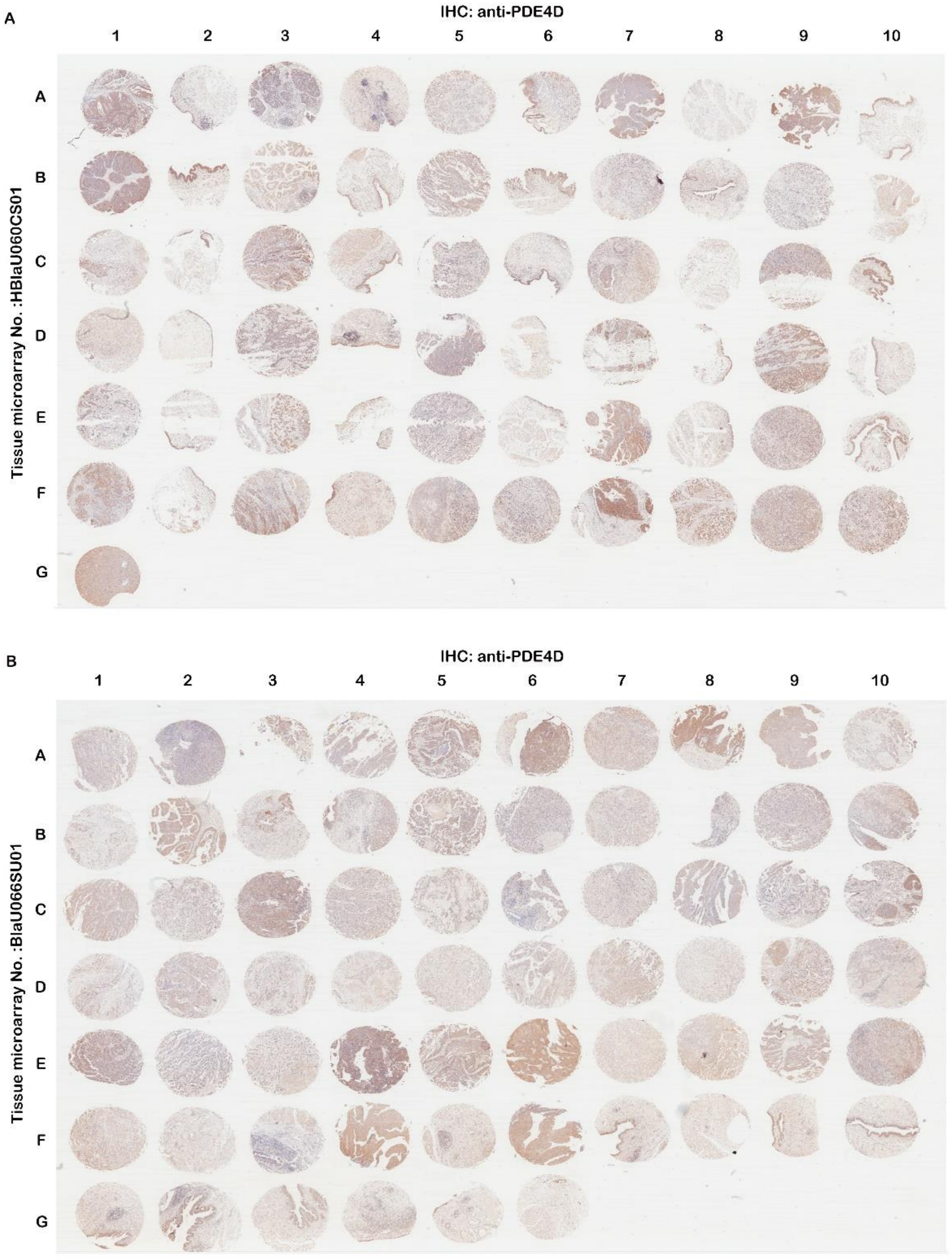

Figure S9: (A-B) Immunohistochemistry images of two tissue microarray chips (No. HBlaU060CS01 **[A]** and No. HBlaU066Su01**[B]**) for PDE4D expression in the bladder tumor tissues and adjacent normal bladder tissues.
