# Supplementary material for: Inhibition of TPL2 by interferon-α suppresses bladder cancer through activation of PDE4D
Source: J Exp Clin Cancer Res. 2018 Nov 27;37:288. doi: 10.1186/s13046-018-0971-4 (PMC6260752; doi:10.1186/s13046-018-0971-4)
Supplement: Supplementary file 10 — Table S1. The differences analysis of the PDE4D and the p-TPL2 expressions between in bladder cancer tissues and in adjacent normal tissues. Table S2. Statistical analysis of the correlations among PDE4D expression, p-TPL2 expression and clinicopathological parameters. (PDF 110 kb) [file 13046_2018_971_MOESM10_ESM.pdf]

Supplementary Table 1 The differences analysis of the PDE4D and the p-TPL2 expressions between in bladder cancer tissues and in adjacent normal tissues

Expression		Mean ± Std. Deviation	P value	Z value
PDE4D	Cytoplasm (tumor)	1.663 ± 0.643	**0.009**	-2.613
	Cytoplasm (tumor-adjacent)	2.118 ± 0.616		
p-TPL2	Cytoplasm (tumor)	6.606 ± 1.903	**0.003**	-2.985
	Cytoplasm (tumor-adjacent)	4.542 ± 2.245		
	Nuclear (tumor)	1.576 ± 1.480	0.261	-1.125
	Nuclear (tumor-adjacent)	1.875 ± 1.296		

The non-parametric Wilcoxon signed-rank test was used for comparison.

Values of $P < 0.05$ were considered statistically significant.

Supplementary Table 2 Statistical analysis of the correlations among PDE4D expression, p-TPL2 expression and clinicopathological parameters

		Characteristic				
		Both sexes	Age (45~75)	Tumor size	TNM stage	Distant metastasis
PDE4D expression	Correlation Coefficient	-.090	**-.356**	.135	**-.241**	.195
	Sig.(2-tailed)	.401	**.001**	.215	**.037**	.096
p-TPL2 expression	Correlation Coefficient	-.060	-.075	-.213	**.538**	-.252
	Sig.(2-tailed)	.781	.729	.330	**.021**	.235

Abbreviations: TNM indicates tumor node metastasis.

Correlation coefficient: ranges from -1 to +1; -1 indicates the strongest possible disagreement and +1 the strongest possible agreement.

Sig. (2-tailed): <0.01 or <0.05 are statistically significant.

Correlation coefficient and Sig. (2-tailed) were obtained from spearman correlation.
