# Supplementary material for: Inhibition of TPL2 by interferon-α suppresses bladder cancer through activation of PDE4D
Source: J Exp Clin Cancer Res. 2018 Nov 27;37:288. doi: 10.1186/s13046-018-0971-4 (PMC6260752; doi:10.1186/s13046-018-0971-4)
Supplement: Supplementary file 11 — Figure S10. (A-B) Immunohistochemistry images of two tissue microarray chips (No. HBlaU060CS01 [A] and No. HBlaU066Su01[B]) for p-TPL2 expression in the bladder tumor tissues and adjacent normal bladder tissues. (PDF 321 kb) [file 13046_2018_971_MOESM11_ESM.pdf]

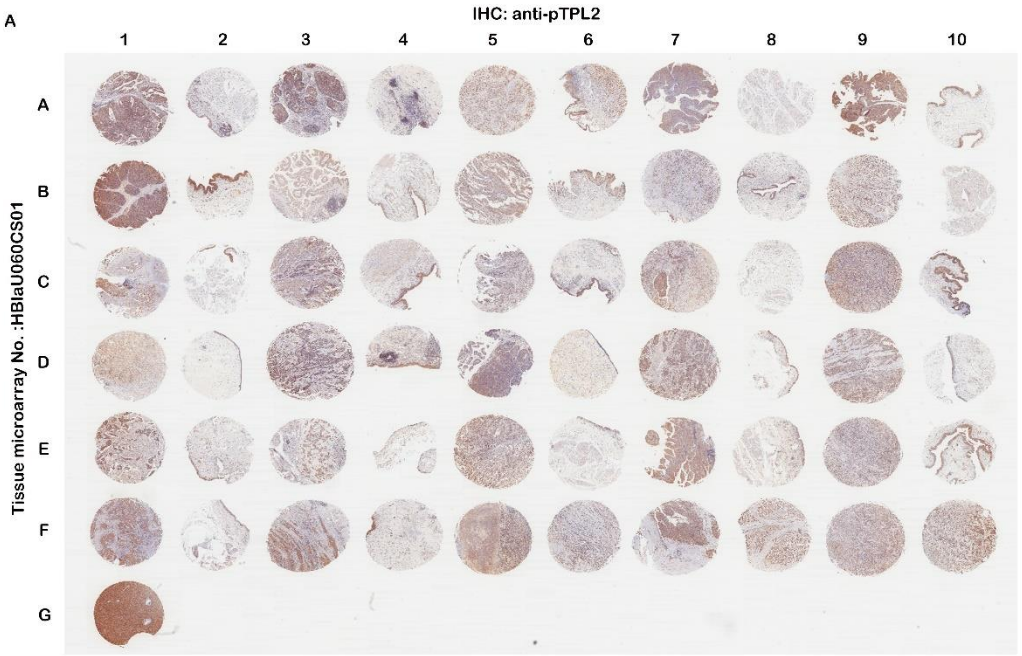

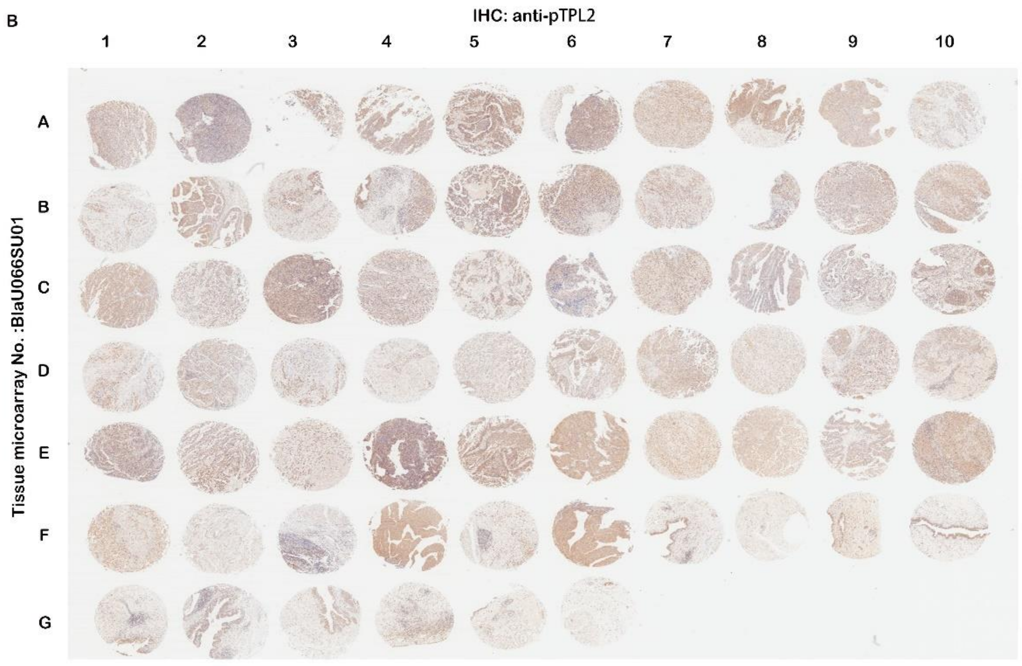

Figure S10: (A-B) Immunohistochemistry images of two tissue microarray chips (No. HBlaU060CS01 **[A]** and No. HBlaU066Su01**[B]**) for p-TPL2 expression in the bladder tumor tissues and adjacent normal bladder tissues.
