# Supplementary material for: Inhibition of TPL2 by interferon-α suppresses bladder cancer through activation of PDE4D
Source: J Exp Clin Cancer Res. 2018 Nov 27;37:288. doi: 10.1186/s13046-018-0971-4 (PMC6260752; doi:10.1186/s13046-018-0971-4)
Supplement: Supplementary file 12 — Figure S11. The survival curves of patients that correspond to each of the clinicopathologic features. All data were obtained from the tissue microarray chips that were used in this study. The survival curves were calculated by the Kaplan-Meier method and analyzed by the log-rank test. (PDF 316 kb) [file 13046_2018_971_MOESM12_ESM.pdf]

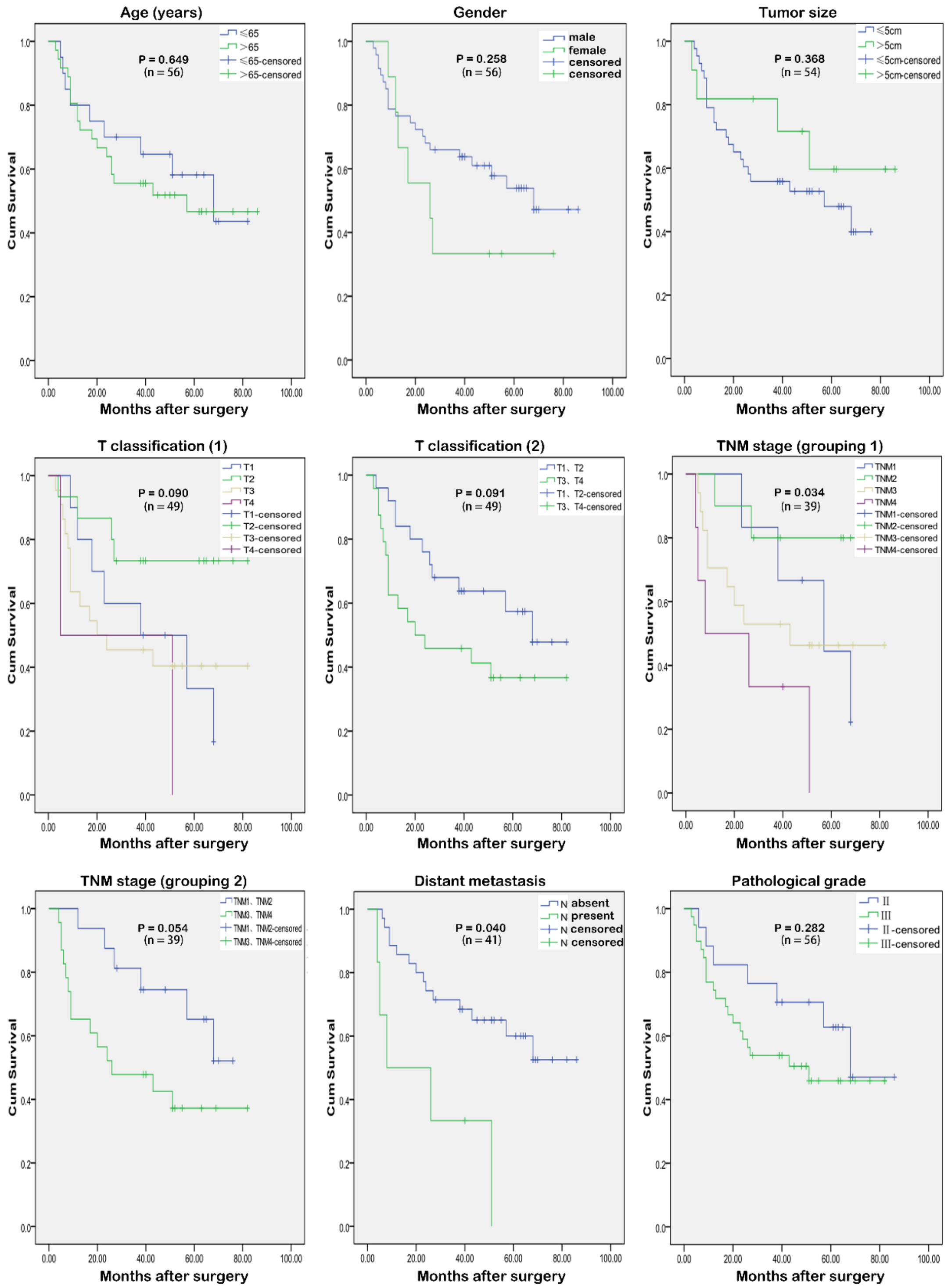

Figure S11: The survival curves of patients that correspond to each of the clinicopathologic features. All data were obtained from the tissue microarray chips that were used in this study. The survival curves were calculated by the Kaplan-Meier method and analyzed by the log-rank test.
