# Supplementary material for: Inhibition of TPL2 by interferon-α suppresses bladder cancer through activation of PDE4D
Source: J Exp Clin Cancer Res. 2018 Nov 27;37:288. doi: 10.1186/s13046-018-0971-4 (PMC6260752; doi:10.1186/s13046-018-0971-4)
Supplement: Supplementary file 13 — Figure S12. The relationships of bladder cancer with the expression of PDE4 family members. (A-C) Compared with PDE4A and PDE4C, the mRNA levels of PDE4B showed a significant down-regulation in bladder tumor than in bladder normal tissue. The data were analyzed by unpaired Wilcoxon test for significance. Values of P < 0.01 were considered statistically significant. All RNA-seq data were obtained from The Cancer Genome Atlas. (https://cancergenome.nih.gov/). (PDF 317 kb) [file 13046_2018_971_MOESM13_ESM.pdf]

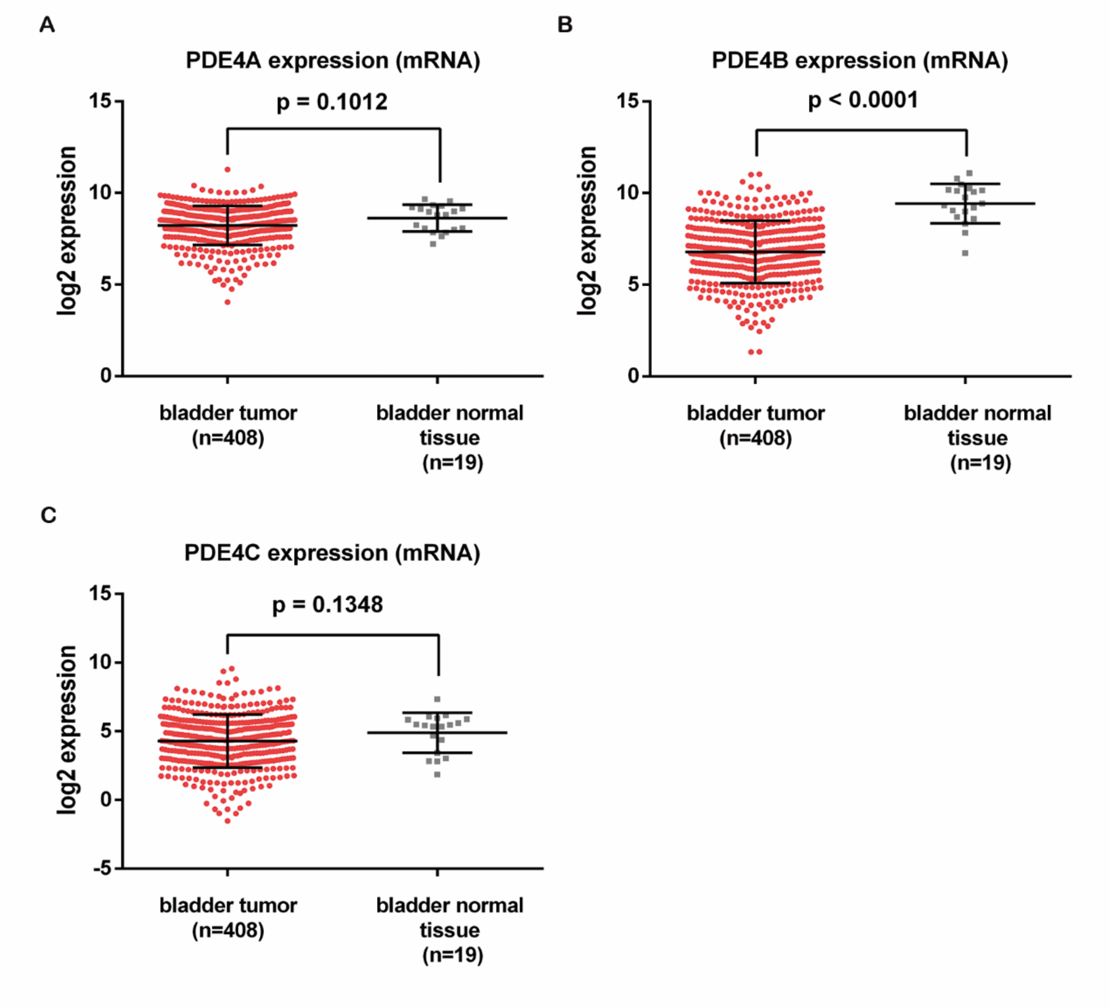

Figure S12: The relationships of bladder cancer with the expression of PDE4 family members. **(A-C)** Compared with PDE4A and PDE4C, the mRNA levels of PDE4B showed a significant down-regulation in bladder tumor than in bladder normal tissue. The data were analyzed by unpaired Wilcoxon test for significance. Values of $P < 0.01$ were considered statistically significant.

All RNA-seq data were obtained from The Cancer Genome Atlas.

(https://cancergenome.nih.gov/).
