# Supplementary material for: Inhibition of TPL2 by interferon-α suppresses bladder cancer through activation of PDE4D
Source: J Exp Clin Cancer Res. 2018 Nov 27;37:288. doi: 10.1186/s13046-018-0971-4 (PMC6260752; doi:10.1186/s13046-018-0971-4)
Supplement: Supplementary file 14 — Figure S13. The relationships of bladder cancer with the expression of PDE4 family members and TPL2. (A-D) The mRNA levels of PDE4D showed the most significant down-regulation in muscle-invasive bladder cancer (MIBC) when compared with the other three PDE4 family members (PDE4A, 4B and 4C). (E) No significant differences of total TPL2 mRNA levels were found among bladder mucosa, NMIBC and MIBC. All data were obtained from the Oncomine database (Lee Bladder Dataset; J Clin Oncol 2010/06/01) and analyzed by unpaired Wilcoxon test. Values of P < 0.01 were considered statistically significant. (PDF 226 kb) [file 13046_2018_971_MOESM14_ESM.pdf]

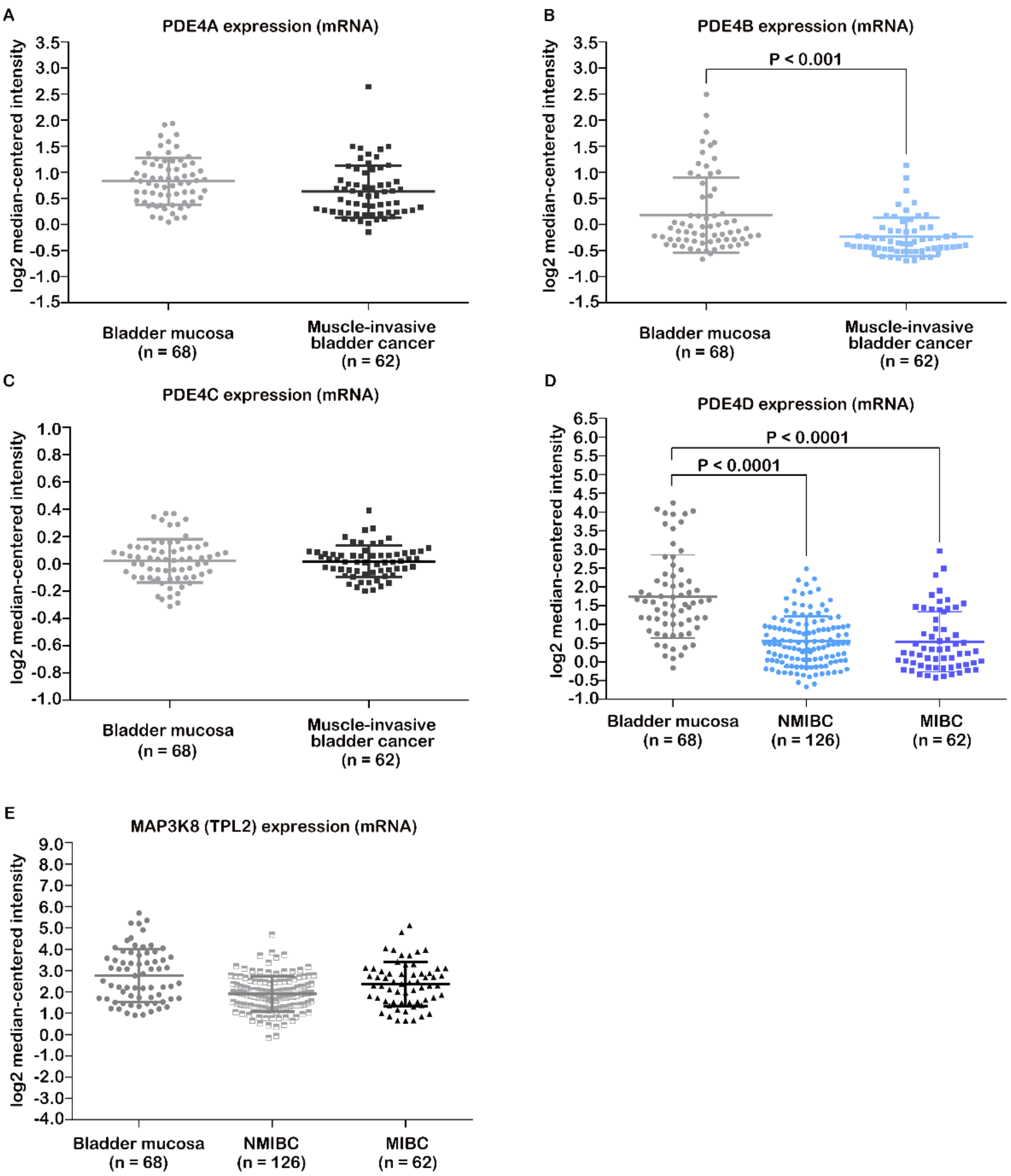

Figure S13: The relationships of bladder cancer with the expression of PDE4 family members and TPL2. **(A-D)** The mRNA levels of PDE4D showed the most significant down-regulation in muscle-invasive bladder cancer (MIBC) when compared with the other three PDE4 family members (PDE4A, 4B and 4C). **(E)** No significant differences of total TPL2 mRNA levels were found among bladder mucosa, NMIBC and MIBC. All data were obtained from the Oncomine database (Lee Bladder Dataset; J Clin Oncol 2010/06/01) and analyzed by unpaired Wilcoxon test. Values of $P < 0.01$ were considered statistically significant.
